# Observations of cross scale energy transfer in the inner heliosphere by Parker Solar Probe

**DOI:** 10.1007/s41614-022-00097-x

**Published:** 2022-11-23

**Authors:** Tulasi N. Parashar, William H. Matthaeus

**Affiliations:** 1 School of Chemical and Physical Sciences, Victoria University of Wellington, Gate 7, Kelburn Parade, Kelburn, Wellington, 6012 New Zealand; 2 Department of Physics and Astronomy, University of Delaware, Sharp Laboratory, Newark, Delaware 19711 USA

## Abstract

The solar wind, a continuous flow of plasma from the sun, not only shapes the near Earth space environment but also serves as a natural laboratory to study plasma turbulence in conditions that are not achievable in the lab. Starting with the Mariners, for more than five decades, multiple space missions have enabled in-depth studies of solar wind turbulence. Parker Solar Probe (PSP) was launched to explore the origins and evolution of the solar wind. With its state-of-the-art instrumentation and unprecedented close approaches to the sun, PSP is starting a new era of inner heliospheric exploration. In this review we discuss observations of turbulent energy flow across scales in the inner heliosphere as observed by PSP. After providing a quick theoretical overview and a quick recap of turbulence before PSP, we discuss in detail the observations of energy at various scales on its journey from the largest scales to the internal degrees of freedom of the plasma. We conclude with some open ended questions, many of which we hope that PSP will help answer.

## Introduction

Since the advent of the space race, the importance of space weather and space environment in general has increased in our lives. The solar wind that shapes this space environment has been a subject of intensive study ever since its prediction and first measurements (Parker 1958; Neugebauer and Snyder 1966). Million degree hot corona is responsible for the acceleration of the solar wind and its eventual escape from the sun into the interplanetary medium. The solar wind, like most naturally occurring as well as man made plasmas, is turbulent in nature (Krommes 2002; Tsytovich 2016; Yamada et al. 2008; Coleman 1968; Matthaeus and Goldstein 1982; Bruno and Carbone 2013). The solar wind, being easily accessible through many space missions, serves as a natural laboratory for studying plasma turbulence in situ. These measurements have allowed testing and refinement of plasma turbulence theories, which are relevant for many other astrophysical systems such as the interstellar medium (Cordes et al. 1985; Falceta-Gonçalves et al. 2014; Armstrong et al. 1995), accretion disks (Balbus and Hawley 1998; Abramowicz and Fragile 2013), and the intracluster medium (Schuecker et al. 2004; Churazov et al. 2012; Mohapatra et al. 2020).

Turbulence is believed to be an important player in heating the solar corona (Hendrix and Van Hoven 1996; Matthaeus et al. 1999; Cranmer et al. 2007). The subsonic coronal plasma accelerates to become supersonic at a few solar radii and eventually super Alfvénic at around $\sim 10-20 R_\odot$. Beyond this Alfvén critical region, the strong coronal magnetic field loses control of the plasma. The shears introduce Kelvin Helmholtz like dynamics, creating large scale roll ups in the solar wind (DeForest et al. 2016; Telloni et al. 2022). These roll-ups push the solar wind towards isotropization at the largest scales introducing an important step in its turbulent evolution (Ruffolo et al. 2020). As the solar wind evolves, the turbulence is believed to keep the wind hotter than what is expected from a simple adiabatic expansion (Richardson et al. 1995). The outer scale of turbulence, the Alfvénicity, and the amplitude of turbulent fluctuations all play an important role in the evolution of the solar wind. Accurate understanding of turbulent processes and their evolution is also critical for improving our global heliospheric models (Usmanov et al. 2011; Gamayunov et al. 2012; Sokolov et al. 2013; Oran 2014; Chhiber et al. 2017; van der Holst et al. 2022).

Iconic missions such as Mariner, Voyager, Helios, and Ulysses ushered an era of exploration of macroscopic as well as turbulent properties of the solar wind. We refer the reader to excellent reviews by (Tu and Marsch 1995; Bruno and Carbone 2013; and Verscharen et al. 2019) for a comprehensive view of solar wind turbulence in the heliosphere before PSP. The basic macroscopic properties of the solar wind are well described in the seminal book by (Hundhausen 1972). Parker Solar Probe (Fox et al. 2016), with its state-of-the-art instrumentation and unprecedented close approaches to the sun is enabling hitherto impossible studies of plasma turbulence close to the sun. In this review paper we discuss how PSP has enhanced our understanding of the turbulent transfer of energy across scales in the inner heliosphere.

We start by describing a phenomenology of scale to scale spectral transfer from energy containing to kinetic scales in Sect.  2. In Sect. 3 we discuss the findings from prior missions such as Voyager, Helios, and Ulysses on the evolution of turbulence in the heliosphere. In Sect.  4 we discuss the new findings enabled by the Parker Solar Probe mission before concluding with a summary of the findings and potential future directions in Sect. 5.

## Turbulent scale-to-scale transfer of energy

Fully developed turbulence is ideally characterized (Batchelor 1970) by an input of energy at some large scales, which is then conservatively transferred to progressively smaller scales in the inertial range. The cascaded energy is eventually converted to internal energy at the dissipative scales (Tennekes and Lumley 1972). The large scale dynamics of plasmas are well described by magnetohydrodynamic description down to fairly small scales (Wu et al. 2013; Karimabadi et al. 2013; Wan et al. 2016). However, in kinetic plasmas, the hydrodynamic notion of a single dissipative scale is replaced by a multitude of smaller scales including the inertial lengths and gyro-radii of protons and electrons, the Debye length, and other hybrid scales. The nature of the cascade modifies at some of these scales, rendering the hydrodynamic cascade picture to be relatively simpler in comparison. More sophisticated theories of turbulence are needed to describe the nature of turbulence at kinetic scales (Schekochihin et al. 2009; Boldyrev et al. 2013; Eyink 2018). Even with the lack of a well accepted kinetic plasma turbulence theory, we can study the transfer of energy across scales in a quantitative way, down to kinetic scales and smaller with appropriate methods. We now describe a phenomenology of such a transfer from the largest scales to kinetic scales and into the internal degrees of freedom.

***Energy at large scales:*** The energy at the largest scales is input by direct sources or by large scale instabilities and is subsequently cascaded down to smaller scales (Biskamp 2003). In hydrodynamics, the turbulent cascade adjusts in such a way as to balance the energy input at the largest scales by dissipation at the small scales (De Karman and Howarth 1938). This von Kármán Howarth description of the decay when generalized to MHD can be written as (Hossain et al. 1995; Wan et al. 2012; Bandyopadhyay et al. 2018)

1 $$\begin{aligned} \epsilon _\pm = -C_\pm \frac{Z_\pm ^2 Z_\mp }{\uplambda _\pm } \end{aligned}$$

where $\epsilon _\pm = \frac{d Z_\pm ^2}{dt}$ is the decay rate for $Z_\pm =\langle |{\mathbf {z}}_\pm |^2\rangle$ with the Elsässer variables defined as $\mathbf {z}_\pm = \mathbf {v} \pm \mathbf {b}/\sqrt{\mu _0 \rho }$ with $\mathbf {v}$, $\mathbf {b}$, and $\rho$ being the fluctuating velocity, magnetic field, and density, and $\uplambda _\pm$ the energy containing scales. The proportionality constant $C_\pm$ can depend on a lot of conditions such as the Reynolds number *Re*, cross helicity $\sigma _c$, and the ratio of thermal to magnetic pressures $\beta =8\pi \mu _0nk_BT/B^2$ (McComb et al. 2015; Matthaeus et al. 2016; Bandyopadhyay et al. 2018).

The von Kármán Howarth similarity, although a simplified large scale description, describes the balance of large scale energy input and dissipation really well not only in hydrodynamics but also in kinetic plasmas down to very small scales (of the order of a few ion inertial scales $d_i$) (Wu et al. 2013; Parashar et al. 2015). In the solar wind the von Kármán Howarth similarity has been recently shown to be applicable to the magnetic field fluctuations (Roy et al. 2021). As discussed in the Sect.  4, PSP has allowed an examination of the balance between energy input and heating rate.

***Energy in the inertial range:*** Assuming isotropy, homogeneity, constancy of scale-to-scale energy transfer rate $\epsilon$, and locality of transfer in the scale space, Kolmogorov (Kolmogorov 1941) identified the power spectrum of hydrodynamic turbulence to be $E(k) = C \epsilon ^{2/3}k^{-5/3}$ (K41), where *E*(*k*) is the energy density in wave-number *k* and $\epsilon$ is assumed to be constant across scales and uniform in space. The K41 phenomenology when extended to MHD predicts spectral slopes varying between $k^{-5/3}$ and $k^{-3/2}$ (Kraichnan 1965; Goldreich and Sridhar 1995; Verma 1999; Zhou et al. 2004; Boldyrev 2005; Lithwick et al. 2007; Chandran 2008; Beresnyak and Lazarian 2008; Perez and Boldyrev 2009). This scaling has been observed in hydrodynamic turbulence behind a grid (Champagne 1978), Earth’s magnetosheath (Parashar et al. 2018; solar wind Kiyani et al. 2015), interstellar medium (Fraternale et al. 2019), and the intracluster medium (Schuecker et al. 2004). If one considers non-uniform dissipation, intermittency emerges while minimally affecting the isotropic form of the spectral law (Kolmogorov 1962; Politano and Pouquet 1995; Verma 2004).

The cascade of energy from large to small scales in the inertial range is quantitatively described, under the assumptions of homogeneity, time stationarity, and isotropy, by the so called third order law. For the conservative part of the cascade, in hydrodynamics, the third order structure function is related to decay rate by $\langle \delta u_\ell ^3 \rangle = - \frac{4}{5}\epsilon \ell$, where $\ell$ is the lag and $\delta u$ is the magnitude of a velocity increment computed at lag $\ell$ (Pope 2000). This third order law was generalized to incompressible MHD by Politano and Pouquet (1998).

2 $$\begin{aligned} Y^\pm (\ell ) = \langle \delta \mathbf {z}^\mp |\delta \mathbf {z}^\pm |^2\rangle = \frac{4}{3} \epsilon ^\pm \ell \end{aligned}$$

where $\ell$ is the lag at which the increment $\delta \mathbf {z}^\pm (\mathbf {x},{\boldsymbol{\ell }}) = \mathbf {z}^\pm (\mathbf {x}+{\boldsymbol{\ell }})-\mathbf {z}^\pm (\mathbf {x})$ is computed, and $\langle \ldots \rangle$ denote appropriate averaging. The incompressible third order law can further be generalized to include more physics in the form of anisotropy (Podesta 2008), compressibility (Andrés and Sahraoui 2017; shears Wan et al. 2009; Hall physics Galtier 2008), some combination of such effects (Ferrand et al. 2021), or be generalized to electron MHD (Galtier 2008). This von Kármán Howarth Yaglom Politano Pouquet (KHYPP) law or Politano-Pouquet (PP) law and many of its extensions to Hall/compressible MHD have been used to measure and test the cascade rates in simulations, solar wind, and Earth’s magnetosheath (Sorriso-Valvo et al. 2007; MacBride et al. 2008; Marino et al. 2008; Boldyrev and Perez 2009; Osman et al. 2011; Verdini et al. 2015; Hellinger et al. 2018; Bandyopadhyay et al. 2020). The cascade rates have also been compared to the expected rates of plasma heating in the solar wind to test if turbulence can account for heating of the solar wind (see discussion in Sects. 3 and 4).

The cascade of energy is actually even more complicated in models as simple as compressible MHD where a compressive cascade proceeds in parallel to a magnetic cascade (Aluie 2011). There exists a small scale ‘decoupled range’ where the magnetic energy and kinetic energy cascades proceed conservatively with the same cascade rate Bian and (Aluie 2019). In this picture the exchange between kinetic and magnetic fluctuations happens at relatively large scales in the inertial range. There are also suggestions that magnetic reconnection, and in some situations large scale instabilities, could potentially bypass the cascade and transfer energy directly into the kinetic range and into internal degrees of freedom (Squire et al. 2017; Franci et al. 2017; Kunz et al. 2020).

The third order law approach becomes cumbersome with the addition of more physics. Moreover the accuracy of this approach depends on the terms retained in the fluid model. An alternative approach to studying scale-to-scale transfer of energy in the fully kinetic limit is to apply scale filtering techniques to the Vlasov equation (Yang et al. 2017); Eyink 2018; Camporeale et al. 2018; Cerri and Camporeale 2020). Starting with the Vlasov equation, applying scale filtering techniques, one arrives at (Yang et al. 2017, 2022)

3 $$\begin{aligned} \partial _t \tilde{{\mathcal {E}}}_\alpha ^f + \nabla \cdot \mathbf {J}_\alpha ^u= & {} - \Pi _\alpha ^{uu}-\Phi _\alpha ^{uT}-\Lambda _\alpha ^{ub} \nonumber \\ \partial _t \bar{{\mathcal {E}}}^m + \nabla \cdot \mathbf {J}^b= & {} - \sum _\alpha \Pi _\alpha ^{bb} + \sum _\alpha \Lambda _\alpha ^{ub} \end{aligned}$$

**Fig. 1:**
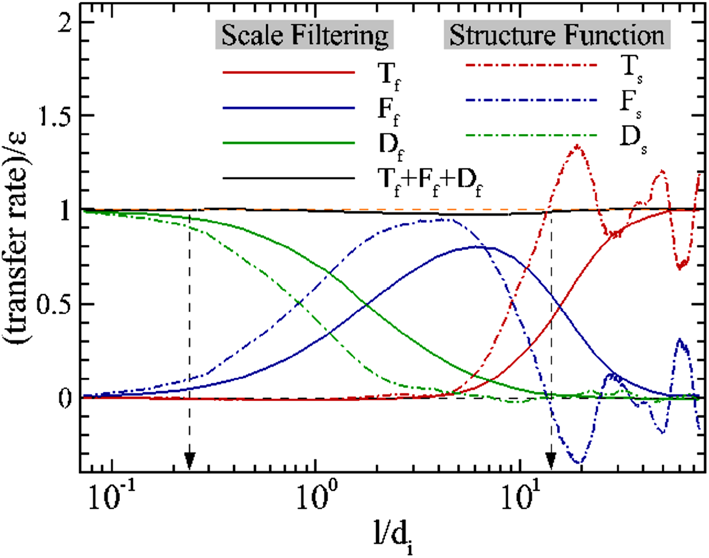
Scale filtered energy fluxes (solid lines) compared to transfer terms from von Kármán Howarth equations generalized to incompressible Hall MHD for a fully kinetic 2.5D simulation of plasma turbulence. The scale filtered energy equations show good numerical energy conservation at all scales while energy conservation has to be imposed on the von Kármán Howarth equations given the lack of kinetic and compressive physics. (Reproduced with permission from Yang et al. 2022)

where $\bar{{phantom{a}}}$ represents a scale filtered quantity and $\tilde{{phantom{a}}}$ represents a density weighted filtered quantity. $\tilde{{\mathcal {E}}}_\alpha ^f$ is the filtered fluid flow energy, $\bar{{\mathcal {E}}}^m$ is the filtered electromagnetic energy, $\mathbf {J}_\alpha ^u$ and $\mathbf {J}^b$ are spatial transport terms, $\Pi _\alpha ^{uu}$, and $\Pi _\alpha ^{bb}$ are the subgrid scale fluxes, $\Phi _\alpha ^{uT}$ is the rate of flow energy conversion to internal energy through pressure strain interactions (see the discussion of energy at kinetic scales below), and $\Lambda _\alpha ^{uT}$ is the rate of energy conversion from electromagnetic fluctuations into fluid flow through filtered $\mathbf {j}_\alpha \cdot \mathbf {E}$. For details of these equations see (Yang et al. 2022).

The Eqs. 3 can be combined to write:

4 $$\begin{aligned} \underbrace{\partial _t \left<\sum _\alpha {\widetilde{\mathcal{E}}}^{f}_{\alpha } + {\overline{\mathcal{E}}}^m\right>}_{T_f-\epsilon } =-\underbrace{\left<\sum _\alpha \left( {\Pi }^{uu}_{\alpha } + {\Pi }^{bb}_{\alpha }\right) \right>}_{F_f}- \underbrace{\left<\sum _\alpha {\Phi }^{uT}_{\alpha }\right>}_{D_f} . \end{aligned}$$

with $T_f-\epsilon$, $F_f$, and $D_f$ representing the decay of energy, the inertial range fluxes, and “dissipation” respectively. These quantities can be directly compared to the generalized von Kármán Howarth equations as shown in Fig. 1. The solid lines represent scale filtered quantities, and the dashed lines correspond to equivalent terms in the von Kármán Howarth equations. The structure function approach seems to achieve a range reminiscent of an inertial range where the inertial range flux is comparable to the decay rate $\epsilon$, while the scale filtered flux remains short of $\epsilon$ even at its peak. However, the scale filtered equations show energy conservation, within numerical error, across scales, giving a quantitative handle on flow of energy across scales in an accurate manner. The von Kármán Howarth equations contain varied limits of physics based on the model for which they are written (Hellinger et al. 2018) and hence energy conservation has been imposed to estimate the surrogate dissipation (shown as a green dot-dashed line). The situation is even more dramatic when the bandwidth available for the cascade is small (e.g. in 3D fully kinetic simulations or the Earth’s magnetosheath). See Yang et al. (2022) for more detailed discussion of these issues. In a recent preprint Hellinger et al. (2022) have included pressure strain interactions to modify the von Kármán equations. This modification extends the range of validity of these equations down to sub-proton scales implying the important role played by pressure-strain interactions in the kinetic range energy transfer; see also Yang et al. (2022).

***Energy transfer at kinetic scales:*** At the ion kinetic scales, some of the energy is removed into heating the ions, and the rest of it cascades down to smaller scales, eventually dissipating at electron scales. Heating of plasma^1^ can potentially happen in many ways including wave-particle interactions such as Landau damping (Hollweg 1971; Chen et al. 2019), cyclotron resonances (Hollweg and Isenberg 2002; Kasper et al. 2013), magnetic pumping (Dawson and Uman 1965; Lichko et al. 2017), and stochastic heating (Chandran et al. 2010; Xia et al. 2013; Mallet et al. 2019; Cerri et al. 2021; Martinović et al. 2021). In the stochastic heating picture, particles experiencing large electric fluctuation changes at their gyro scales can get stochastic kicks perpendicular to the mean magnetic field changing their magnetic moment. This effect, that depends on turbulent fluctuation amplitude at the proton gyro scale, gets enhanced near intermittent structures such as current sheets (Chandran et al. 2010; Xia et al. 2013; Mallet et al. 2019). Moreover, landau damping has also been shown to occur in or near current sheets (TenBarge et al. 2013).

Many factors such as plasma $\beta$, turbulence amplitude, proton-electron temperature ratio, Alfvénicity etc. can potentially regulate the fraction of energy going into heating the ions (Wu et al. 2013; Hughes et al. 2014; Matthaeus et al. 2016). A simplified view (Matthaeus et al. 2016) proposes that the ratio of the local nonlinear time at the ion scales to the cyclotron time is an important factor in deciding the partitioning of energy between ions and electrons. If the kinetic scale nonlinear time is comparable to or smaller than the proton cyclotron time, significant nonlinear evolution of turbulent magnetic fluctuations happens within a gyro-period and hence the protons can get significant stochastic kicks, leaving a smaller amount of energy to cascade down to electron scales and eventually heat them. Such dependence of relative proton-electron heating has been shown to hold in simulations as well as recently in MMS data (Matthaeus et al. 2016).

**Fig. 2:**
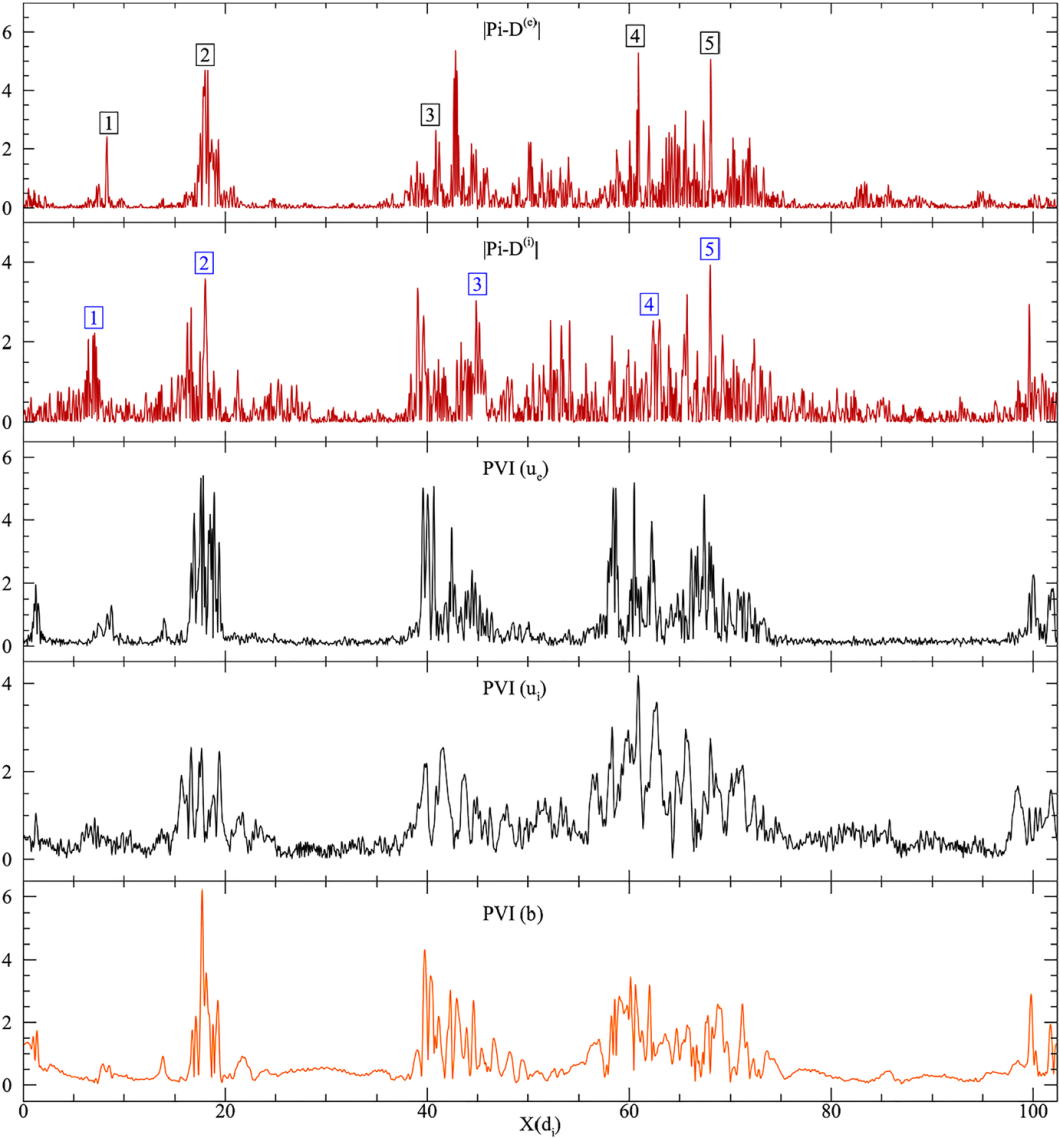
Pi-D, the pressure strain interaction, is active near current sheets and is spatially more correlated with velocity strains than current. (Reproduced with permission from Yang et al. 2017.)

Although the exact processes responsible for heating the ions can vary from one scenario to the other, the mathematical terms responsible for the transfer are fairly straightforward to understand. The transfer of energy from electromagnetic fields and bulk flow energy into internal degrees of freedom happens via a collisionless generalization of viscosity. The equations for time evolution of electromagnetic energy $E^m$, fluid flow kinetic energy $E^f_\alpha$, and internal energy $E^{th}_\alpha$ can be written in a straightforward manner from the Vlasov Maxwell set of equations as (Braginskii 1965; Yang et al. 2017)

5 $$\begin{aligned} \partial _t E^{f}_\alpha + \nabla \cdot \left( E^{f}_\alpha {\varvec{u}}_\alpha + {\varvec{P}}_\alpha \cdot {\varvec{u}}_\alpha \right)= & {} \left( {\varvec{P}}_\alpha \cdot \nabla \right) \cdot {{\varvec{u}}}_\alpha + {\varvec{j}}_\alpha \cdot {\varvec{E}}, \nonumber \\ \partial _t E^{th}_\alpha + \nabla \cdot \left( E^{th}_\alpha {\varvec{u}}_\alpha + {\varvec{h}}_\alpha \right)= & {} -\left( {\varvec{P}}_\alpha \cdot \nabla \right) \cdot {\varvec{u}}_\alpha , \nonumber \\ \partial _t E^{m} + {\frac{c}{4\pi }} \nabla \cdot \left( {\varvec{E}} \times {\varvec{B}} \right)= & {} -{\varvec{j}} \cdot {\varvec{E}}, \end{aligned}$$

where the subscript $\alpha =e, i$ represents the species, ${\varvec{P}}_\alpha$ is the pressure tensor, ${\varvec{h}}_\alpha$ is the heat flux vector, ${\varvec{j}}=\sum _{\alpha } {\varvec{j}}_\alpha$ is the total electric current density, and ${\varvec{j}}_\alpha =n_\alpha q_\alpha {\varvec{u}}_\alpha$ is the electric current density of species $\alpha$. The divergence terms do not convert energy from one form into another. They simply transport energy in its current form. The ${\varvec{j}} \cdot {\varvec{E}}$ term is responsible for transfer of energy from electromagnetic fields into bulk flow and the $-\left( {\varvec{P}}_\alpha \cdot \nabla \right) \cdot {\varvec{u}}_\alpha = P^{(\alpha )}_{ij} \nabla _i\, u^{(\alpha )}_j$ term (called PS for short) is responsible for transferring energy from bulk fluid motions into internal degrees of freedom (Del Sarto et al. 2016; Yang et al. 2017).

The pressure tensor $P^{(\alpha )}_{ij}$ can be separated into a trace and a traceless part by defining $P^{(\alpha )}_{ij} = p_\alpha \delta _{ij} + \Pi ^{(\alpha )}_{ij}$ where $p_\alpha = \frac{1}{3} P^{(\alpha )}_{jj}$ and, $\Pi _{ij} = P_{ij} - p\delta _{ij}$. The stress tensor $S^{(\alpha )}_{ij}=\nabla _i u^{(\alpha )}_j$ can be similarly decomposed $S^{(\alpha )}_{ij} = \frac{1}{3}\theta _\alpha \delta _{ij} + D^{(\alpha )}_{ij} + \Omega ^{(\alpha )}_{ij}$ where $\theta =\nabla \cdot {{\mathbf {u}}}$, $D^{(\alpha )}_{ij} = \frac{1}{2}\left( \nabla _i u^{(\alpha )} _j + \nabla _ju^{(\alpha )}_i\right)$, and $\Omega ^{(\alpha )}_{ij} = \frac{1}{2}\left( \nabla _i u^{(\alpha )} _j - \nabla _ju^{(\alpha )}_i \right)$. With these decompositions, the pressure stress interaction separates as $\left( \mathbf {P}_\alpha \cdot \nabla \right) \cdot \mathbf{u}_\alpha = p^{(\alpha )} \theta ^{(\alpha )} + \Pi ^{(\alpha )}_{ij}D^{(\alpha )}_{ij}$. The first term is responsible for heating/cooling due to compressions/rarefactions and is typically abbreviated as $p\theta$. The second part, typically called Pi-D, reduces to the familiar viscous heating term (Huang 2008) in the highly collisional limit. The pressure tensor is symmetric and hence only the symmetric stresses of bulk velocity interact with the traceless part of the pressure tensor to achieve the conversion into internal energy.

Kinetic activity, including the heating of the ions via the Pi-D channels takes place intermittently *near* strong current sheets (Servidio et al. 2012, 2014; Franci et al. 2016; Del Sarto et al. 2016; Parashar and Matthaeus 2016). Sheared magnetic fields produce strong current sheets, which in turn develop vortex quadrupoles near them (Matthaeus 1982; Parashar and Matthaeus 2016). Although the vorticity is the antisymmetric part of the velocity strain tensor, the vortices are stretched into sheet like structures in the large Reynolds number limit, creating symmetric parts of the velocity strain tensor. This symmetric part contracts with the traceless pressure tensor to transfer energy from bulk turbulent motions into internal degrees of freedom (Del Sarto et al. 2016; Yang et al. 2017). The pressure strain interaction has been shown to be an effective description of plasma heating in simulations as well as in magnetosheath data (Yang et al. 2017; Sitnov et al. 2018; Matthaeus et al. 2020; Bandyopadhyay et al. 2020).

The intermittent sites near which the dissipation occurs can be identified using partial variance of increments (PVI), defined as ${\mathcal {I}}=|\Delta \mathbf {b}(t,\Delta t)|/\sqrt{\langle |\mathbf {b}(t,\Delta t)|^2\rangle }$ where $\Delta \mathbf {b}(t,\Delta t) = \mathbf {b}(t+\Delta t)-\mathbf {b}(t)$ (Greco et al. 2018), or via the unaveraged kernel of the third order law (Eq. 2), also called Local Energy Transfer rate (LET) (Sorriso-Valvo et al. 2018). Figure 2 shows cuts of Pi-D for ions and electrons from a 2.5D fully kinetic simulation along with PVI computed from velocities of ions and electrons and magnetic field (Yang et al. 2017). The locations of enhanced dissipation, identified by spikes in Pi-D, are clustered near large PVI values. Large PVI values have been shown to be strongly correlated with hotter ions in the solar wind (Osman et al. 2011), and with higher fluxes of energetic particles (Tessein et al. 2013).

An imbalanced cascade, with different powers in the $\mathbf {z}^+$ and $\mathbf {z}^-$ fluctuations and relevant for example for inner heliospheric conditions, could modify the cascade at ion kinetic scales affecting the ion heating rates and resulting cascade to smaller scales. The ‘helicity barrier’ inhibits the cascade of energy to scales smaller than proton kinetic scales, resulting in a build-up of energy at the proton scales. This build-up of proton scale kinetic energy can result in generation of cyclotron waves, which can heat the protons perpendicularly (Squire et al. 2022). This enhanced energy dissipation at ion kinetic scales can result in very steep spectra just below ion kinetic scales (approaching $k^{-4}$), eventually returning to the more familiar $k^{-8/3}$ close to electron scales.

**Fig. 3:**
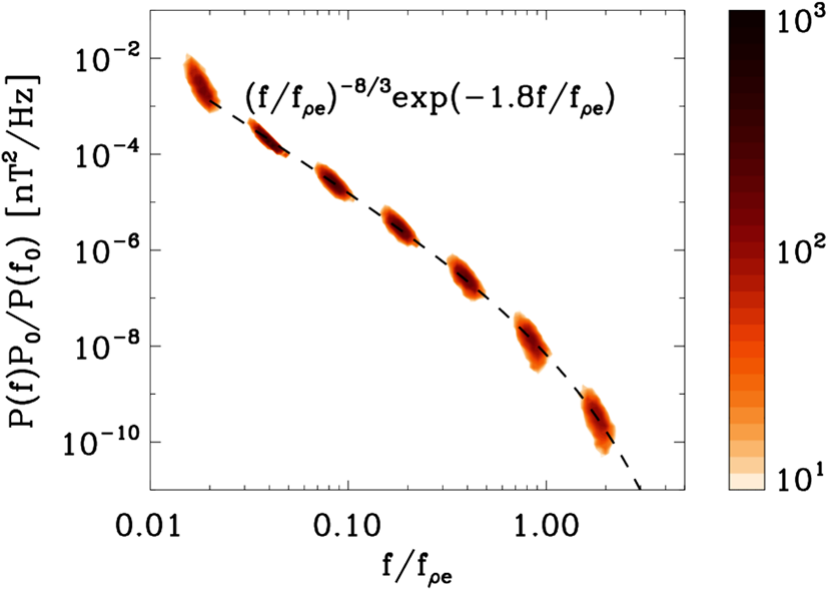
Magnetic energy spectra in the inner heliosphere computed using SCM data from the Helios spacecraft. The spectra show a power law superposed with exponential decay of magnetic fluctuations at electron scales. (Reproduced with permission from Alexandrova et al. (2021))

Close to electron scales, the magnetic energy spectra are exponentially damped. Figure 3 shows magnetic energy spectra computed using magnetic field measurements from the search coil magnetometer (SCM) on Helios. The 3344 individual spectra have been rescaled by their amplitude at roughly 20 electron gyro-periods. The colours represent 2D histograms with darker colours representing more points. The spectra show a power-law behaviour with $f^{-8/3}$ superposed with exponential decay at electron scales indicating strong damping of magnetic fluctuations (Alexandrova et al. 2012; TenBarge et al. 2013; Arrò et al. 2021).

**Fig. 4:**
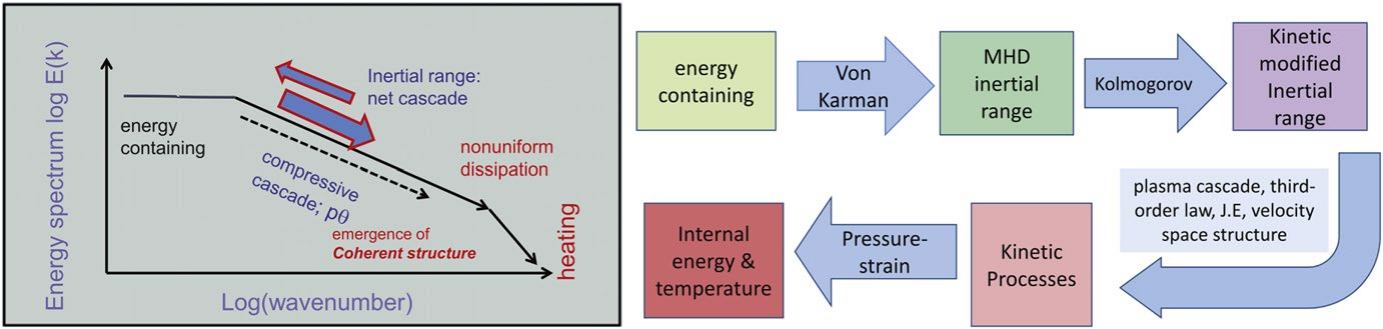
A schematic representation of the flow of energy from large to kinetic scales in kinetic plasma turbulence (Reproduced with permission from Matthaeus et. al. ApJ 2020).

Based on the above considerations, an overall view of the cascade of energy from large to small scales emerges to be as follows (see Fig.  4 and Matthaeus et al. (2020) for an in-depth discussion): The energy containing scales input energy at the von Kármán rate into the inertial range. In the inertial range a cascade of energy to smaller scales via incompressible as well as compressive cascades transfers energy conservatively to smaller scales. The cascade also generates intermittent structures where pressure strain interactions transfer energy into internal degrees of freedom. The remaining part of the energy is transferred down to smaller scales where kinetic effects become dominant and dissipation ends the cascade.

## Turbulence in the heliosphere before PSP

The dynamics of turbulence and its effects on the evolution of the solar wind have been studied in-depth since the late 60s. The observations from Mariners, Voyagers, Helios, and Ulysses revealed how the turbulent power, Alfvénicity, power spectra, spectral anisotropy, turbulent cascade, and intermittency evolve with heliocentric distance. For comprehensive reviews of solar wind turbulence we refer the reader to (Tu and Marsch 1995; Bruno and Carbone 2013; Verscharen et al. 2019).

Among the pioneering and landmark early studies of turbulence in the interplanetary medium, an important example is the work of Coleman (1968). This study synthesized analysis of Mariner 2 data, taking in to account earlier observations (Holzer et al. 1966; Coleman 1966) as well as the important suggestion (Sturrock and Hartle 1966) that energy in waves or turbulence may be responsible for heating the corona. Coleman (1968) developed this idea by postulating that the physical processes leading this heating would be governed approximately by ideas from classical hydrodynamic turbulence theory Chandrasekhar (1949) adapted to plasma in the approach of Kraichnan (1965). This lead to a heating rate due to the cascade that was found to be reasonably in accord with observed temperatures at 1 AU. Several decades of research have elaborated on these ideas.

**Fig. 5:**
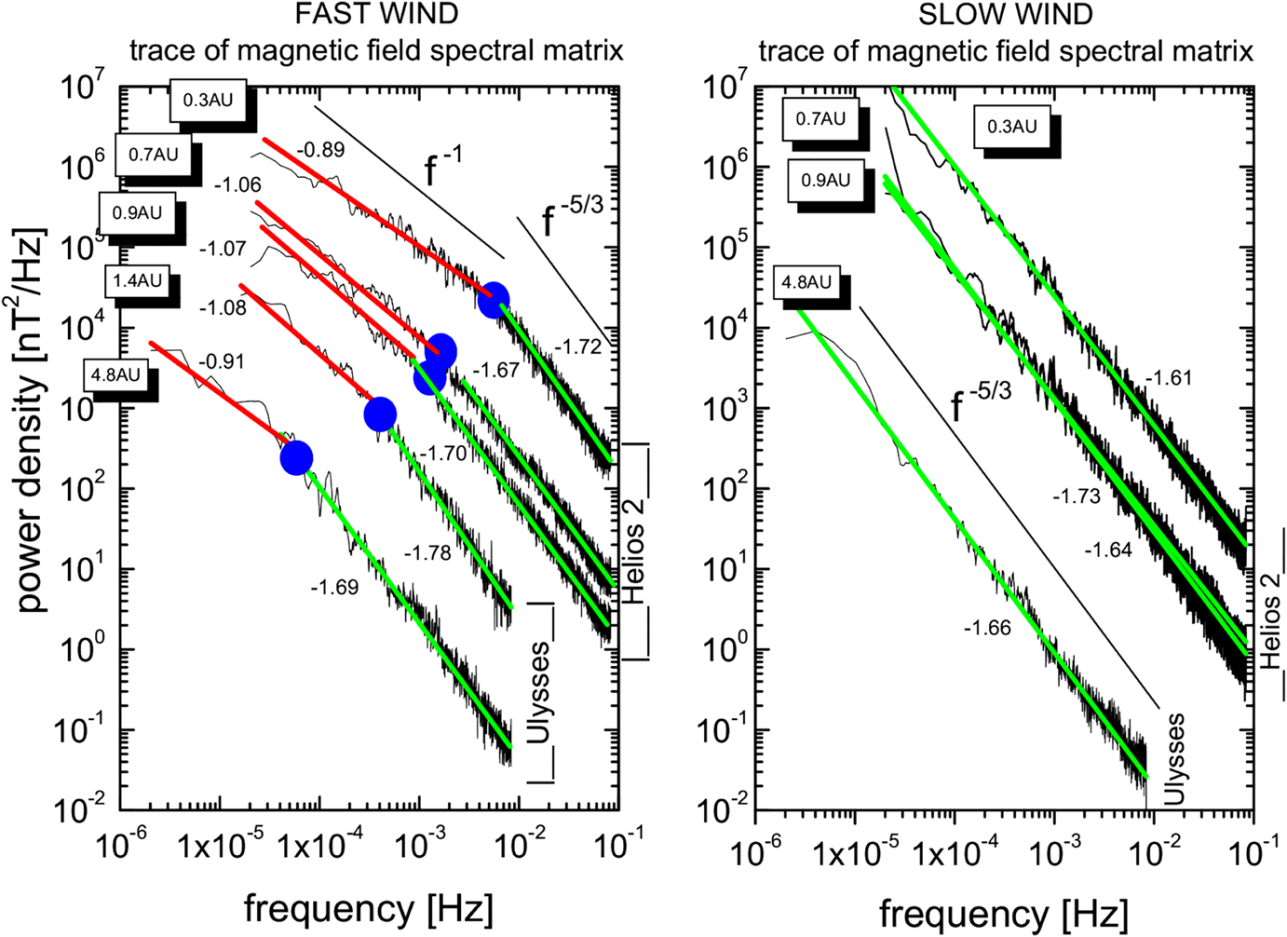
Magnetic field spectra for various heliocentric distances computed from Helios and Ulysses data. Left panel shows fast wind cases and the right panel shows slow wind cases. Spectral power decreases with increasing heliocentric distance regardless of the speed of the wind. The fast wind spectra show a transition from Kolmogorov like $f^{-5/3}$ to $f^{-1}$ at large scales. The break frequency moves to larger scales with increasing heliocentric distance. The slow wind does not show such a transition. (Reproduced with permission from Bruno and Carbone (2013))

As the solar wind expands, the turbulent power decreases with heliocentric distance (Belcher and Burchsted 1974). From earlier inner heliospheric observations (Roberts et al. 1990), one may argue that this decrease is consistent with WKB theory (Verma and Roberts 1993). However it is also consistent with a driven, dissipative expanding MHD system (Zank et al. 1996) which gives very similar radial profiles for appropriate parameter choices. Interestingly, as also shown in Zank et al. (1996), the solutions are *not* consistent with an undriven dissipative turbulence system, which would provide power at 1 AU that is less than what is observed. The observed decreasing total power reflects as well in the reduced spectral densities with increasing heliocentric distance (Bavassano et al. 1982; Horbury and Balogh 2001; Bruno et al. 2009). Describing this evolution of the spectrum and understanding the somewhat subtle physical effects that enter this description has been the subject of intensive study, even until the time of this writing.

Figure 5 shows power spectra for magnetic field computed from Helios and Ulysses data (Bruno et al. 2009). The left panel shows trace magnetic power spectra for fast wind intervals and the right panel shows the same for slow wind intervals at various heliocentric distances. Two important features are clearly identified. The fast wind spectra show a break at large scales and transition from $f^{-5/3}$ to $f^{-1}$ at some large scale. The location of this spectral break shifts to lower frequencies or larger scales as the solar wind expands in the heliosphere. The break frequency follows a power law decrease of $R^{-1.5}$ with heliocentric distance. The power law exponents for this radial trend show some yet unexplained variability. This will be discussed below as well as in Sect. 4. Secondly, the slow wind spectra do not show a transition to an $f^{-1}$ regime, potentially owing to the more advanced state of evolution of the observed slow wind.

The first theoretical studies to attempt a description of the radial evolution of heating (Hollweg 1986; Hollweg and Johnson 1988) and the radial evolution of the spectral shapes (Tu et al. 1984; Tu 1988) made major steps towards merging the ideas of turbulence theory with spatial transport modeling of radial evolution of solar wind properties, a theory classically exemplified by WKB theory (Hollweg 1974). It was soon recognized that refinements of these approaches were required for greater veracity, including the crucial development of *non-WKB transport* theory (Marsch and Tu 1989; Zhou and Matthaeus 1989) and transport theory to describe the turbulence fluctuations at *energy containing scales* (Matthaeus et al. 1994) that feed energy into the inertial range (Dmitruk et al. 2002; Cranmer and Van Ballegooijen 2005; Verdini and Velli 2007; Chandran and Hollweg 2009; Van Ballegooijen et al. 2011; Perez and Chandran 2013; Van Ballegooijen and Asgari-Targhi 2016; Zank et al. 2018; Chandran and Perez 2019).

The energy containing scale, identified by the correlation length $\Lambda = \tau _{corr}V_{sw}$, increases with increasing heliocentric distance (Smith et al. 2001; Ruiz et al. 2014). Measurements from Voyager and Ulysses show the increase to follow $R^{0.45}$, in contrast to the expectation of break frequency variation of $R^{-1.5}$. Along with the highly non-adiabatic behavior of the proton temperature (Richardson et al. 1995; Smith et al. 2001), the observed variation of correlation scale is a strong indication of the macroscopic influence of active turbulence evolution in the interplanetary medium.

**Fig. 6:**
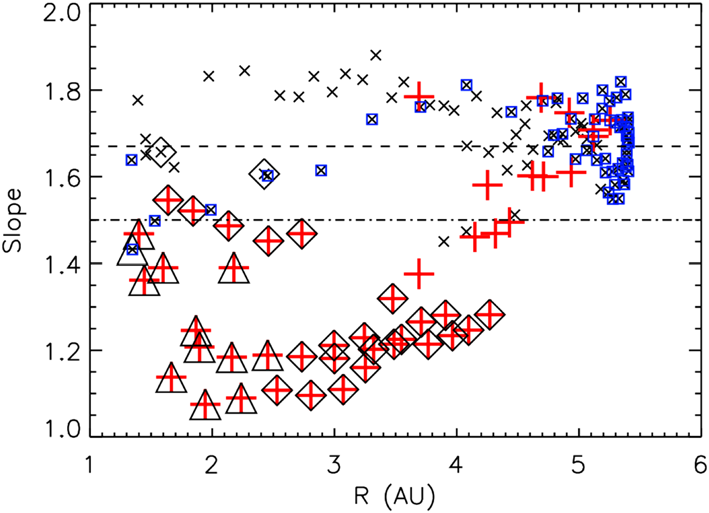
Slopes of velocity spectra from Ulysses spacecraft as a function of heliocentric distance. Red plus signs represent intervals with wind speed greater than 675 km/s, all other points are black crosses. Intervals with signed Alfvénicity between 0.33 and 0.5 are enclosed in diamonds and the ones with high Alfvénicity are enclosed in triangles. Points within 20$^\circ$ of the ecliptic are enclosed in blue squares. (Reproduced with permission from Roberts (2010))

The characteristics of the plasma velocity fluctuations are also indicative of turbulence and of turbulence evolution as the solar wind ages. The velocity spectra evolve with heliocentric distance towards a state that is closer to a Kolmogorov-like spectral index. Figure 6 shows slopes of velocity wave-number spectra from Ulysses data as a function of heliocentric distance (Roberts 2010). The slopes were computed using power law fits to the velocity spectra in the $10^{-5}$ Hz to $10^{-4}$ Hz range. Red pluses represent very fast wind with speed $> 675$ km/s and black crosses represent rest of the intervals. These intervals show a significant scatter in spectral slopes between 1-2 AU but show a gradual rise of the slope from $\sim -1.1$ to $\sim -5/3$ in the outer heliosphere. A reason behind this could potentially be that the $10^{-5} - 10^{-4}$ Hz fitting range might be in the energy containing range in the inner heliosphere and in the inertial range as the wind expands and the break point moves to lower frequencies (see for example Fig. 5). The points enclosed in blue squares are within 20$^\circ$ of the ecliptic and hence directly comparable with results from other missions such as Helios and PSP. The slopes near the ecliptic vary from $-3/2$ to $-5/3$ as heliocentric distance increases. As interesting as this may be, one must recall that the standard Kolmogorov theory applied to MHD does not make a specific universal prediction about the velocity spectrum itself, but rather, in the usual sense, for the total incompressive energy spectrum.

**Fig. 7:**
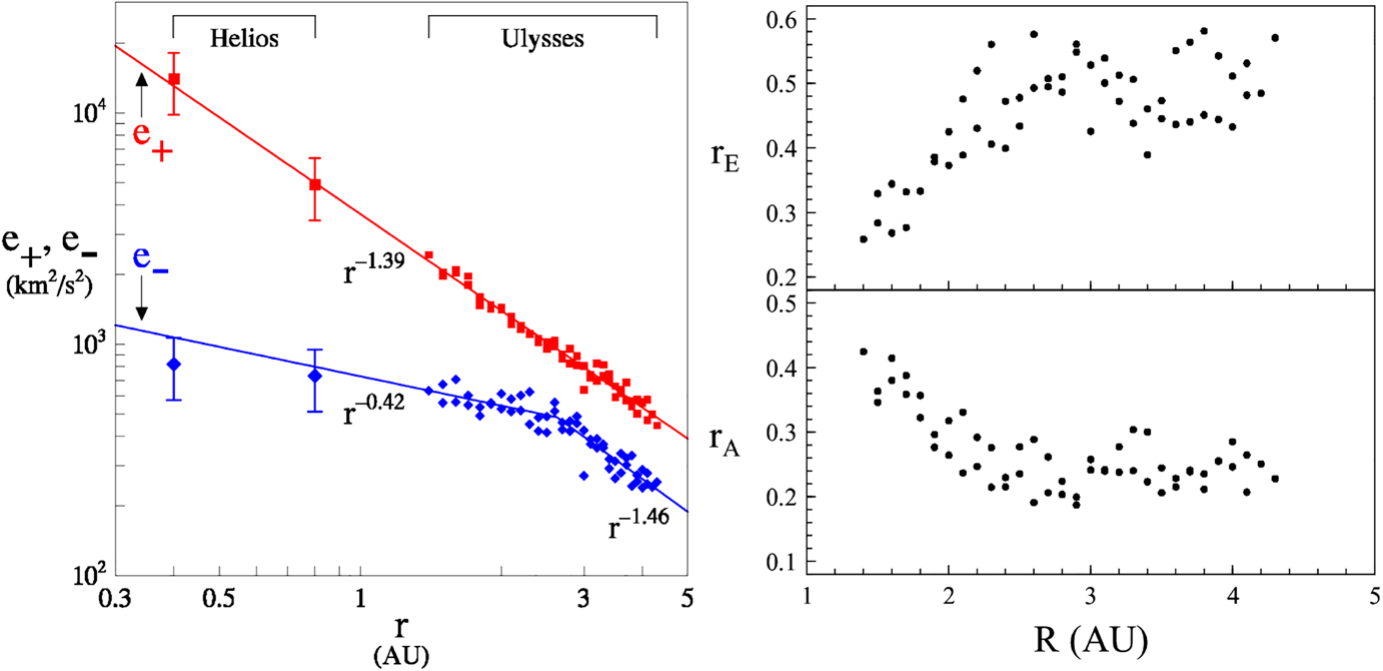
Radial variation of Elsässer variances from Helios and Ulysses (left panel), Elsässer ratio (top right panel) and Alfvén ratio (bottom right panel). The outward Elsässer variable decays faster than the inward Elsässer variable until roughly 2.5 AU after which the outward and inward Elsässer variables show roughly the same energy while the Alfvén ratio fluctuates near 0.2. (Reproduced with permission from Bruno and Carbone (2013))

The turbulence is in an “imbalanced” high cross helicity state in the inner heliosphere. It is highly Alfvénic with the outward propagating Elsässer field dominating the energy budget. This is usually argued to be a consequence of the launching of outward-propagating waves in the lower corona and photosphere, along with the potential filtering effect that may occur at the Alfvén critical surface (Only outward waves propagate away.) However it is well known that an admixture of inward-type cross helicity is required to drive an incompressive MHD cascade (Kraichnan 1965). A widely accepted explanation for how the cascade is enabled is that interactions with Alfvén speed gradients, i.e., “reflections,” (Velli et al. 1989; Matthaeus et al. 1999) or, equally well, interaction with shears (Matthaeus et al. 1999) that tap velocity field energy and produces *both* senses of Elasässer propagation, can produce the required flux of inward fluctuations. For a detailed discussion of these ideas see Bruno and Carbone (2013) and the references therein.

High latitude solar wind turbulence shows a smaller inertial range than what is observed in the ecliptic because of the lack of shears (Goldstein et al. 1995), or perhaps weaker shears (Breech et al. 2008). As the solar wind expands, the cross helicity and the Alfvénicity of the solar wind fluctuations decrease (Roberts et al. 1987; Goldstein et al. 1995; Matthaeus et al. 2004; Bavassano et al. 2000). Fig. 7 shows the radial evolution of Elsässer variances (left panel), the ratio of energies contained in the Elsässer variables, called Elsässer ratio in the top right panel, and the Alfvén ratio in the bottom right panel. This quantity is defined as the ratio of energy density in the velocity fluctuations to energy density in magnetic fluctuations, i.e., $r_A = \langle \mathbf{v}^2 \rangle /\langle \mathbf{b}^2 \rangle$. The outward Elsässer energy decreases significantly faster than the inward Elsässer energy out to roughly 2.5 AU beyond which both decrease in a similar fashion. The ratio of the two Elsässer energies increases gradually as the turbulence becomes less Alfvénic; this ratio fluctuates around 0.5 beyond 2.5 AU.

Apart from the variation of cross helicity, there is also systematic radial evolution of the Alfvén ratio. In the inner heliosphere, the inertial range $r_A$ decreases and stabilizes at values around $r_A \approx 1/2$. Like cross helicity, $r_A$ is influenced by both expansion and shear. An equivalent quantity is the *residual energy* defined as $\sigma _r = (\langle \mathbf{v}^2 \rangle - \langle \mathbf{b}^2 \rangle )/(\langle \mathbf{v}^2 \rangle + \langle \mathbf{b}^2\rangle )$, a quantity that is not related to ideal invariants and so is not associated with a conserved spectral flux. Even if it cannot “cascade” in the usual sense, $\sigma _r$ exhibits distinctive properties, such as attaining moderately negative values $\sigma _r \sim -1/3$ in the inertial range of MHD turbulence and in the solar wind over a fairly wide range of parameters. (For observations, see, e.g., Matthaeus and Goldstein 1982.) There have been numerous phenomenological theories developed to describe the behavior of residual energy. See e.g., Stribling and Matthaeus (1991); Müller and Grappin (2004); Boldyrev et al. (2011); Grappin et al. (2016).

**Fig. 8:**
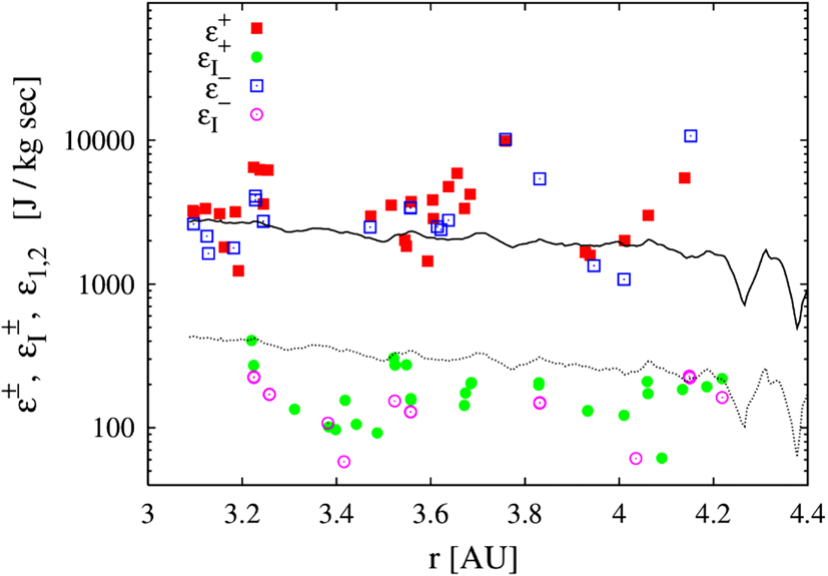
Incompressible as well as compressible cascade rates (defined using density weighted Elsässer variables), computed from Ulysses data, as a function of heliocentric distance. The lines show estimated heating rates for solar wind obtained from temperature profiles. (Reproduced with permission from Carbone et al. (2009))

The temperature of the solar wind drops slower than expected as it expands until the pickup ions introduce a new significant source of energy in its evolution (Marsch et al. 1982; Wang and Richardson 2001; Matteini et al. 2007; Hellinger et al. 2013). The heating rate computed from large scale properties in the solar wind at 1 AU is sufficiently larger than the required heating rate (Vasquez et al. 2007). The nonlinear cascade of energy to smaller scales, measured by the third order law (Eq. 1), has also been established in the solar wind (Sorriso-Valvo et al. 2007; MacBride et al. 2008; Carbone et al. 2009; MacBride et al. 2005; Marino et al. 2012). The incompressible cascade rate, studied in the polar wind using Ulysses data, can provide a significant fraction of energy required to heat the solar wind (Marino et al. 2008). When generalized phenomenologically to include compressibility effects via density weighted Elsässer fields, the cascade rate increases significantly. Figure 8 shows cascade rates computed for both incompressible Elsässer variables as well as density weighted Elsässer variables. The compressive estimates are about an order of magnitude higher and for both estimates follow the heating rate required for heating the solar wind protons (Verma et al. 1995; Vasquez et al. 2007; Marino et al. 2008).

Near kinetic scales the cascaded energy is transferred partially into proton internal energy and partially cascaded down to electron scales. Data from Helios missions have been used to compute the expected stochastic heating (SH) rates in the inner heliosphere (Bourouaine and Chandran 2013; Martinović et al. 2019). The stochastic heating rate appears to be sufficient to heat the solar wind. However, the radial dependence of the stochastic heating rate is very steep ($r^{-2.5}$) and it decreases more rapidly than the expected heating rate. This disparity is larger for the fast solar wind streams. The stochastic heating process is enhanced in the presence of the intermittent structures (Chandran et al. 2010; Xia et al. 2013; Mallet et al. 2019).

In simulations, and in observations at 1 AU, the heating of plasmas has been shown to happen intermittently (Parashar et al. 2011; Osman et al. 2011, 2012; TenBarge et al. 2013; Yang et al. 2017). Until recently there have not been many studies that investigated intermittent heating behaviour in the inner heliosphere; this is discussed more in a later section below. However, the radial evolution of intermittency has been studied in some detail (Bruno et al. 2003; Parashar et al. 2019; Cuesta et al. 2022). The kurtosis at a given time-scale seems to increase with increasing heliocentric distance (Bruno et al. 2003). When plotted as a function of plasma scales (e.g. multiples of proton inertial length $d_p$), the kurtosis drops with increasing heliocentric distance (Parashar et al. 2019; Cuesta et al. 2022). The proton inertial length $d_p$ increases faster than the outer scale of the turbulence in expanding wind. This reduces the bandwidth available for the inertial range cascade, and hence the level of intermittency as identified by scale dependent kurtosis at a given plasma scale.

## Parker Solar Probe observations

Parker Solar Probe was launched in 2018 to study the origins and evolution of the solar wind (Fox et al. 2016). The science objectives of the probe are to “*trace the flow of energy that heats the corona and accelerates the solar wind*”, to “*determine the structure and dynamics of the magnetic fields at the sources of solar wind*”, and to “*explore the mechanisms that accelerate and transport energetic particles*” (Fox et al. 2016). The mission carries four instrument suites: Electromagnetic Fields Investigation (FIELDS) (Bale et al. 2016), Integrated Science Investigation of the Sun (IS$\odot$IS) (McComas et al. 2016), Solar Wind Electrons Alphas and Protons (SWEAP) (Kasper et al. 2016), and Wide-field Imager for Solar Probe (WISPR) (Vourlidas et al. 2016). The data from FIELDS, SWEAP, and IS$\odot$IS have extensively been used to study the origins and evolution of solar wind turbulence and its role in energetic particle dynamics. In this section we discuss PSP’s contributions to our understanding of how the turbulent energy flows from large to small scales in the inner heliosphere. After a brief overview of the turbulence properties (see [190] for an in-depth review), we provide an in-depth discussion of the turbulent transfer of energy from large scales to small scales.

As one approaches closer to the sun, the turbulence becomes more structured. Intermittent patches of reversals in radial magnetic field are embedded in ‘*smoother and less turbulent flow with near radial magnetic field*’ (Bale et al. 2019). These routinely observed feature of the solar wind, also called ‘magnetic switchbacks’ typically show a sharp reversal in the sign of the radial component of the magnetic field (Balogh et al. 1999; Matteini et al. 2014; Borovsky 2016; de Wit et al. 2020). The origins of switchbacks are debated. Proposed mechanisms involve interchange reconnection, in-situ generation by expanding turbulence, and velocity shears (Fisk and Kasper 2020; Squire et al. 2020; Ruffolo et al. 2020). In the shear driven picture, large velocity shears across magnetic flux tubes are dominated by the strong magnetic field in the corona. After the Alfvén critical zone, the magnetic field is not strong and velocity shears can produce “flocculated” roll ups and switchbacks in-situ (DeForest et al. 2016; Chhiber et al. 2018; Ruffolo et al. 2020). A consequence of such shear driven in-situ generation of switchbacks is that their number should drop in the sub-Alfvénic wind inside magnetically dominated corona. Early hints of that are already being observed in the PSP data (Bandyopadhyay et al. 2022; Kasper et al. 2021). A significant amount of literature stemming from PSP observations has focused on comparing and contrasting the nature of turbulence in and outside switchbacks (see e.g. de Wit et al. 2020; Bourouaine et al. 2020; Martinović et al. 2020; Tenerani et al. 2020; McManus et al. 2020; Martinović et al. 2021; Bandyopadhyay et al. 2022 and references therein for a sampling of topics studied in the context of switchbacks by PSP).

**Fig. 9:**
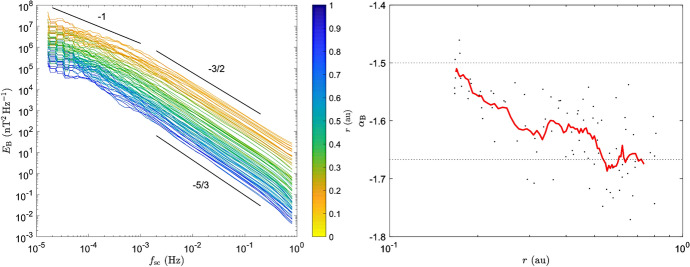
(Left) Magnetic field spectra in the energy containing and MHD inertial range computed from PSP magnetic field data, (Right) Spectral slopes in the inertial range. The spectral slopes show a clear transition from Kolmogorov like to IK like as the radial distance decreases. (Reproduced with permission from Chen et al. (2020).)

The turbulent power, at the energy containing and inertial range scales, increases sunwards, consistent with earlier findings (Belcher and Burchsted 1974; Bavassano et al. 1982; Horbury and Balogh 2001; Bruno et al. 2009; Chen et al. 2020). The inertial range slopes of the magnetic field power spectra gradually shift from Kolmogorov-like $k^{-5/3}$ Kolmogorov (1941) to Irochnikov–Kraichnan like $k^{-3/2}$ (Iroshnikov 1964; Kraichnan 1965) with decreasing radial distance (Chen et al. 2020; Sioulas et al. 2022). Figure 9 shows magnetic field spectra from the first two orbits of PSP in the left panel and the computed slopes in the right panel. Net power in the magnetic fluctuations is seen to rise sunwards. Most of the spectra show a 1/*f* range at the largest scales and the inertial range slopes transition from $-3/2$ in the inner heliosphere to $-5/3$ at 1 AU. Another analysis, using Hilbert Huang Transform (HHT), computed multifractal scalings for magnetic field fluctuations observed by PSP (Alberti et al. 2020). The spectral slopes are confirmed to transition from IK like -3/2 to Kolmogorov like -5/3 at 0.4 AU. The turbulence also shows a transition at this radial distance from monofractal (for $r <0.4$ AU) to multi-fractal nature (for $r > 0.4$ AU). This change from monofractal to multi-fractal behaviour appears to be in contrast with the conclusion that MHD range intermittency has a solar origin and that the in-situ driving is not strong enough to maintain the level of intermittency further away from the Sun (Macek 2012; Wawrzaszek et al. 2019, and references therein). It should however be noted that the latter conclusions were for distances larger than 1 AU. A combined view of the evolution of intermittency might be that the intermittency increases in the inner heliosphere (Alberti et al. 2020, 2022) and decreases beyond 1 AU (Wawrzaszek et al. 2019; Parashar et al. 2019).

The outer scale of the turbulence, as characterized by the correlation time, changes from $\sim 500 s$ at around 0.17 AU to a couple of hours at around 1 AU (Parashar et al. 2020; Chen et al. 2020). The spectral break in Fig. 9 between $f^{-1}$ and $f^{-5/3}$ regimes is seen to shift to lower frequencies with increasing heliocentric distance, consistent with earlier studies (Bavassano et al. 1982; Bruno et al. 2009; Bruno and Trenchi 2014). The large-scale break frequency has been shown to follow a power-law variation with heliocentric distance (Bruno and Carbone 2013; Chen et al. 2020; Wu et al. 2020). The power-law exponent was found to be $\sim 1.1$ by Chen et al. (2020). Although Wu et al. (2020) did not perform a fit to their data, the qualitative behaviour shown by them is similar to that shown by Bruno and Carbone (2013) with an exponent of $\sim 1.5$. Interestingly, these variations are seen in previous studies as well (Horbury et al. 1996; Klein et al. 1992; Bruno and Carbone 2013; Ruiz et al. 2014). The slope found by Chen et al. (2020) is shallower than what was found by Bruno and Carbone (2013) and steeper than those found by Ruiz et al. (2014). Recently, Cuesta et al. (2022) studied the heliocentric variation of the outer scale using data from Helios, Voyager, and three intervals from PSP. The Voyager values are consistent with Ruiz et al. (2014), Helios data show a slightly steeper rise, and the three PSP intervals are placed to indicate a very steep slope in the inner heliosphere. When the directionality, based on the angle between the magnetic field and solar wind velocity, is taken into account, the outer scales show perpendicular anisotropy with $\uplambda _\parallel /\uplambda _\perp \approx 0.75$ at 0.10 AU. These roughly isotropize by 1 AU (Cuesta et al. 2022). The turbulence evolution models (Zank et al. 1996; Matthaeus et al. 1996) predict slopes shallower than -1 and are consistent with Ruiz et al. (2014). A comprehensive study is needed to understand the origins of such differences. At the kinetic scales, the break frequency between the inertial range and sub-proton range also shifts to lower frequencies as $r^{-1.1}$ (Duan et al. 2020). The kinetic scale break frequency is also observed to be closely correlated with the cyclotron frequency along with other parameters such as plasma $\beta$.

Consistent with earlier observations, the Alfvénicity of the fluctuations decreases with heliocentric distance. The cross helicity as well as the energy budget of sunward and outward Elsässer variables, decreases as the wind expands Shi et al. (2021). The dominant outward Elsässer variable decays faster than the sunward Elsässer variable (see Fig. 7, reproduced from (Bavassano et al. 2000), and equivalent figures in Chen et al. (2020)). Some intervals in the inner heliosphere show a decrease in cross helicity across scales, indicating a possibility of strong velocity shears destroying the cross helicity in the inertial range (Parashar et al. 2020). MHD simulations with shears as well as analysis of Helios intervals with shear show a similar reduction in cross helicity at shear sites (Roberts et al. 1992).

**Fig. 10:**
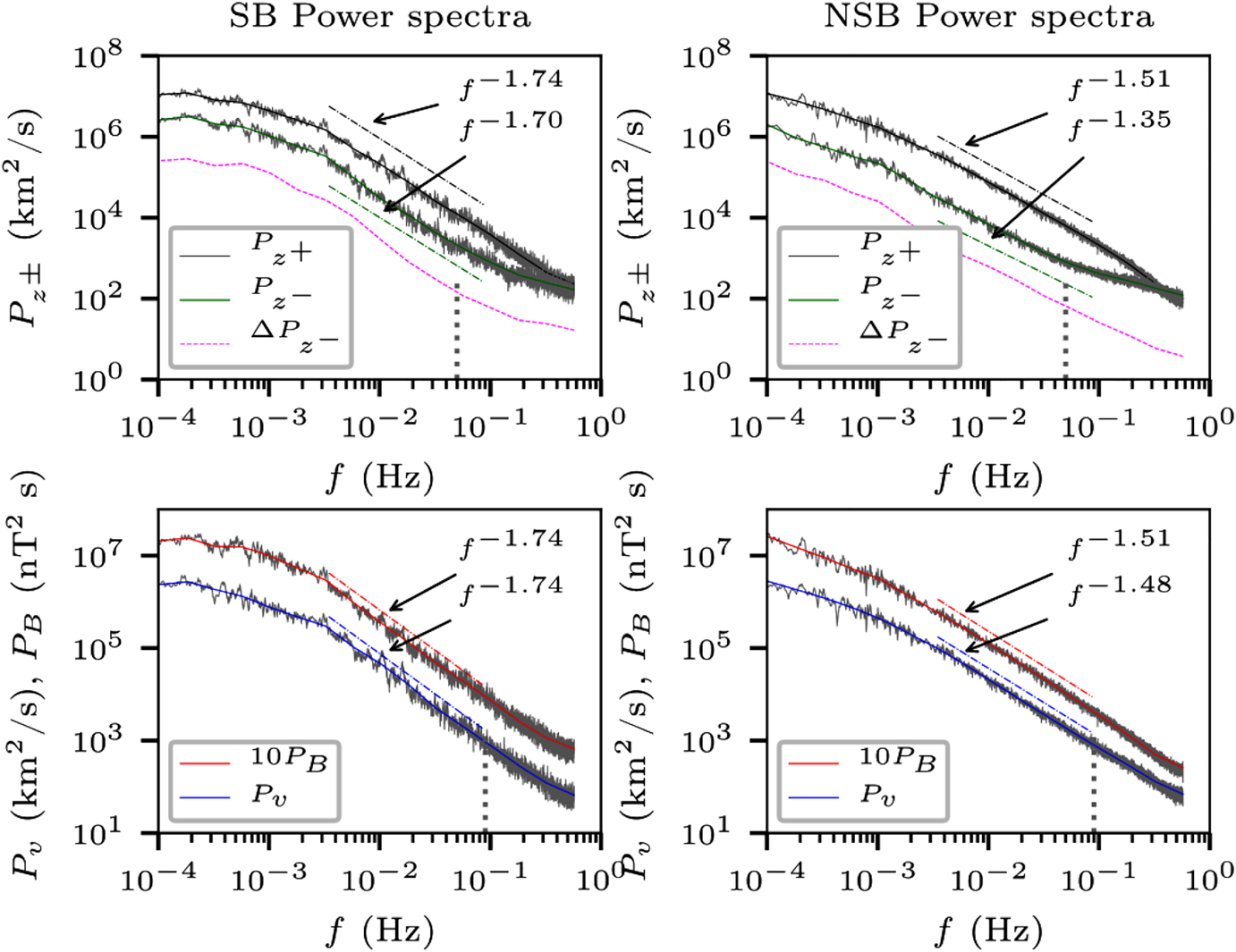
Power spectra for the Elsässer variables, velocity as well as the magnetic field in switchback (SB, left panels) and non switchback (NSB, right panels) intervals. The spectra show Kolmogorov like spectral slopes close to $-5/3$ inside SBs and IK like $-3/2$ in NSB intervals. (Reproduced with permission from Bourouaine et al. (2020))

While many quantities important for turbulence, e.g. the magnitudes of wind speed, temperature, magnetic field and density, remain comparable between SBs and nearby ‘quiet’ regions, some properties of turbulence such as spectral signatures, decay rates, and intermittency properties vary significantly between switchbacks and non-switchback intervals (Bourouaine et al. 2020; Martinović et al. 2021). Figure 10 (from Bourouaine et al. (2020)) shows power spectra in the spacecraft reference frame for intervals with and without switchbacks from PSP’s first encounter (E1) in November 2018. Left panels show Elsässer, velocity, and magnetic field spectra for the switchback interval and the right panels show the same spectra for non-switchback intervals. The spectra for all variables in the SB intervals show Kolmogorov like $f^{-5/3}$ spectra, while the NSB intervals show IK like $f^{-3/2}$ spectra. A potential reason behind this could be intense driving of turbulence by velocity shears that are likely responsible for switchbacks (Ruffolo et al. 2020). The more evolved turbulence in the SB regions produces larger PVI events with higher probability (Martinović et al. 2021).

**Fig. 11:**
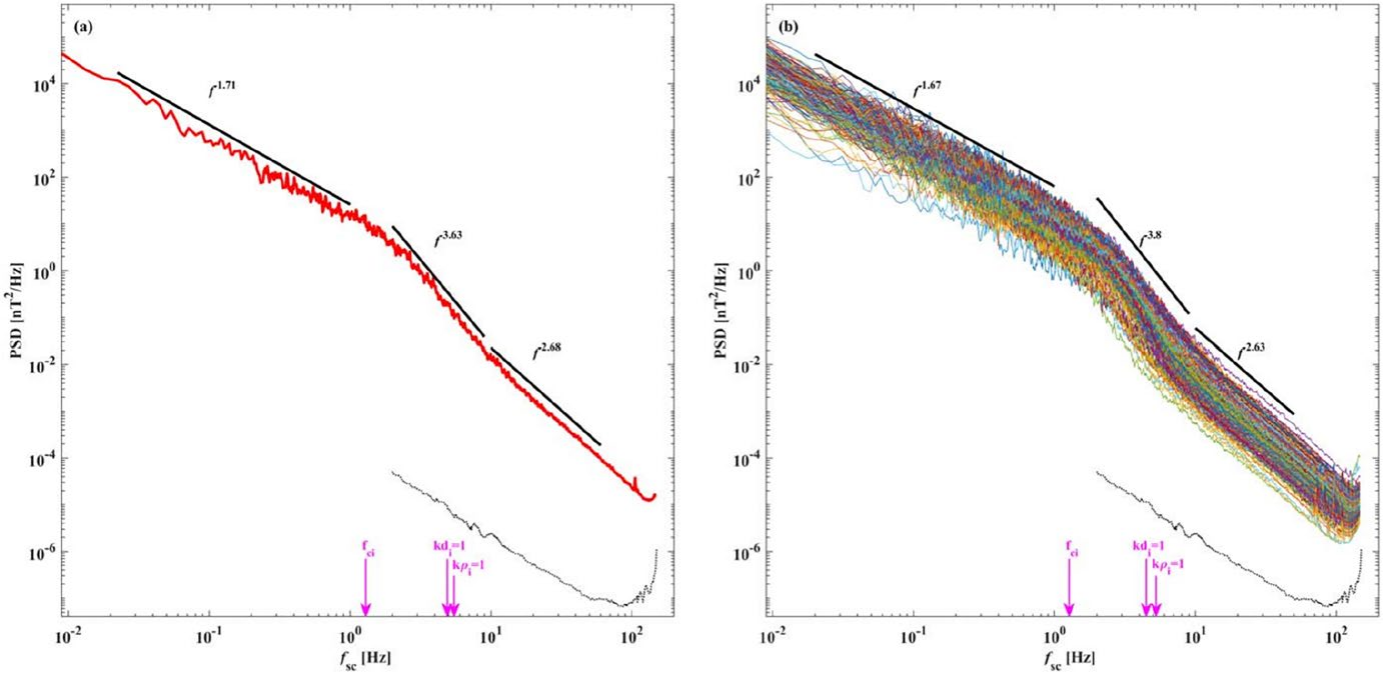
The magnetic field spectra show a steep transition range at the ion kinetic scales. The transition range typically shows a spectral slope of $\sim -4$ before turning to the familiar spectral slope of $\sim -8/3$ at the higher frequencies. (Reproduced with permission from Huang et al. (2021))

Another commonly observed feature of turbulence in the inner heliosphere is the enhanced steepening of magnetic field power spectra near the ion kinetic scales. Figure 11 shows magnetic field spectra computed using the merged Fluxgate magnetometer (FGM) and Search Coil Magnetometer (SCM) data (Bowen et al. 2020a; Huang et al. 2021). The spectra shown are from encounter 1 of PSP, specifically from November 4–7, 2018. A transition range near proton kinetic scales is seen with a slope of $f^{-4}$. This sharp transition indicates a modified cascade or enhanced dissipation at kinetic scales or a combination thereof. The spectra return to $f^{-8/3}$ approaching electron kinetic scales. Such transition range has also been observed at 1 AU in Wind observations (Denskat et al. 1984; Leamon et al. 1999). One of the possible explanations proposed for this transition is the “helicity barrier”, which emerges in imbalanced turbulence and reduces electron dissipation of kinetic Alfvén waves (KAWs) (Meyrand et al. 2021; Squire et al. 2022).

**Fig. 12:**
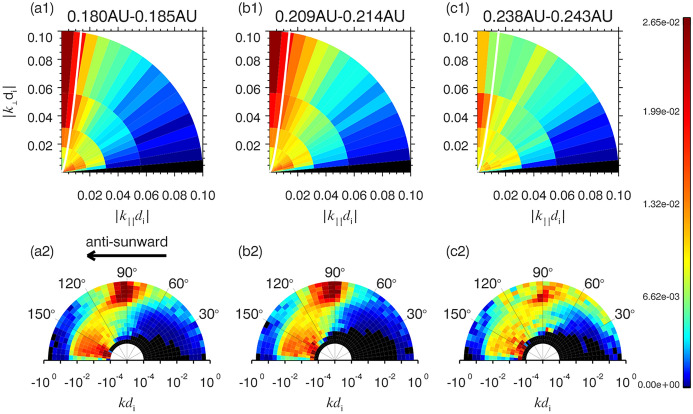
PDFs of wavevectors identified in PSP data during the first encounter of PSP. Top row shows the PDFs in $k_\perp -k_\parallel$ plane and the bottom row shows the data in $k-\theta _{k,B_0}$ plane. Dominance of predominantly parallel wavevectors for large scales and predominantly perpendicular wavevectors for smaller wavenumbers is evident. (Reproduced with permission from Zhu et al. (2020))

As expected for MHD turbulence, the fluctuations are highly anisotropic in nature. Fig 12 shows an example from the first encounter of PSP (Zhu et al. 2020). The top row shows probability distribution functions (PDFs) of identified wave vectors in the $k_\perp - k_\parallel$ plane, and the bottom row shows the PDFs in $k-\theta _{k,B_0}$ plane where $\theta _{k,B_0}$ is the angle between the wavevector and the background magnetic field. The wavenumbers studied were in the MHD regime. In the inertial range, for $kd_i < 0.02$ the fluctuations are predominantly parallel. Towards the tail of the inertial range, for $kd_i > 0.02$, the fluctuations are predominantly perpendicular. Also the outward component of the Alfvénic fluctuations dominates. The fluctuations are still highly anisotropic in the transition and kinetic ranges, with power in perpendicular fluctuations being roughly an order of magnitude higher than the parallel fluctuations (Duan et al. 2021; Zhang et al. 2022; Huang et al. 2022).

In summary, the turbulence observed by PSP shows a lot of structure with interspersed switchbacks and “quiet” Alfvénic intervals. The SBs generally have more evolved turbulence compared to NSB intervals. The power is distributed highly anisotropically in the wavenumber space. The Alfvénicity of the wind decreases with increasing heliocentric distance, and the imbalance of turbulence is likely responsible for a steep transition range near proton kinetic scales. We now discuss the PSP observations of energy transfer across scales.

### Energy at large scales

**Fig. 13:**
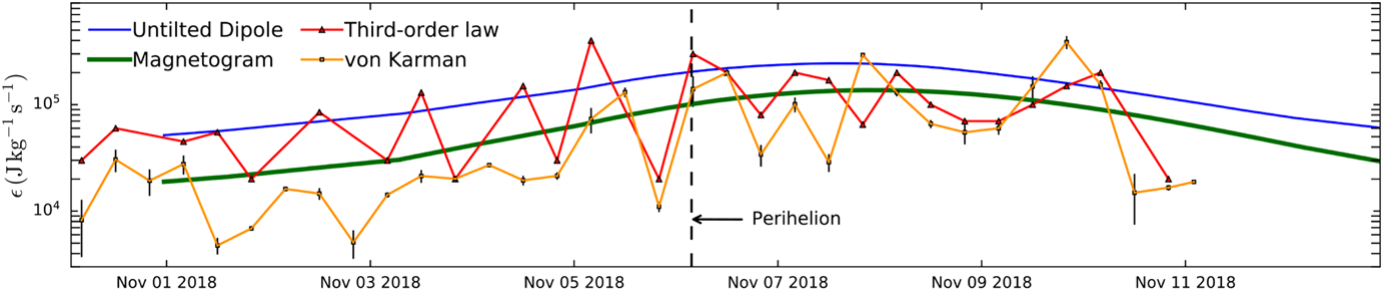
Decay rates from PSP’s first encounter, computed in two ways: squares with orange line show the large scale von Kármán estimate and triangles with red line show the third order law estimates. The thin blue line and thick green line show two global simulations with untilted dipole and the relevant solar magnetogram as inputs for solar magnetic field. (Reproduced with permission from Bandyopadhyay et al. (2020))

The plasma driven at large scales by various solar inputs, in-situ velocity shears, and instabilities receives energy at a rate quantified by the von Kármán decay rate. Figure  13 shows the decay rates computed from encounter 1 of PSP (Bandyopadhyay et al. 2020). The squares with orange line show the von Kármán decay rate based on large scale parameters and the triangles with red line show the energy transfer rates estimated by fitting the third order structure functions following the Politano Pouquet law (see next subsection for more details). The two smooth solid lines show two decay rates computed from two global simulations (Chhiber et al. 2019). The thin blue line is a simulation with sun’s magnetic field represented as an untilted dipole, and the green line is a simulation with the magnetogram from the time of encounter 1. The decay rates increase with decreasing heliocentric distance, changing by more than an order of magnitude within a few solar radii in which the encounter data were collected. The overall decay rates obtained near the perihelion are a couple of orders of magnitude larger than 1000 J Kg$^{-1}$s$^{-1}$ decay rate that is observed at 1 AU (Coburn et al. 2015). The von Kármán decay rates and the third order law estimates match better in the outgoing part of E1, presumably because that was highly Alfvénic slow wind, which might have a higher density of intermittent structures. The global simulations estimate the decay rates reasonably well with the magnetogram simulation overlapping really well with the two estimates from PSP.

The heating rates in the inner heliosphere have also recently been computed from angular broadening studies of Crab nebula (Raja et al. 2021). The angular broadening observations were used to estimate the average density perturbations, which were then converted to velocity fluctuations assuming kinetic Alfvén wave like properties. The decay rates computed from these velocity fluctuation levels are in the same ballpark as the numbers quoted in Bandyopadhyay et al. (2020).

**Fig. 14:**
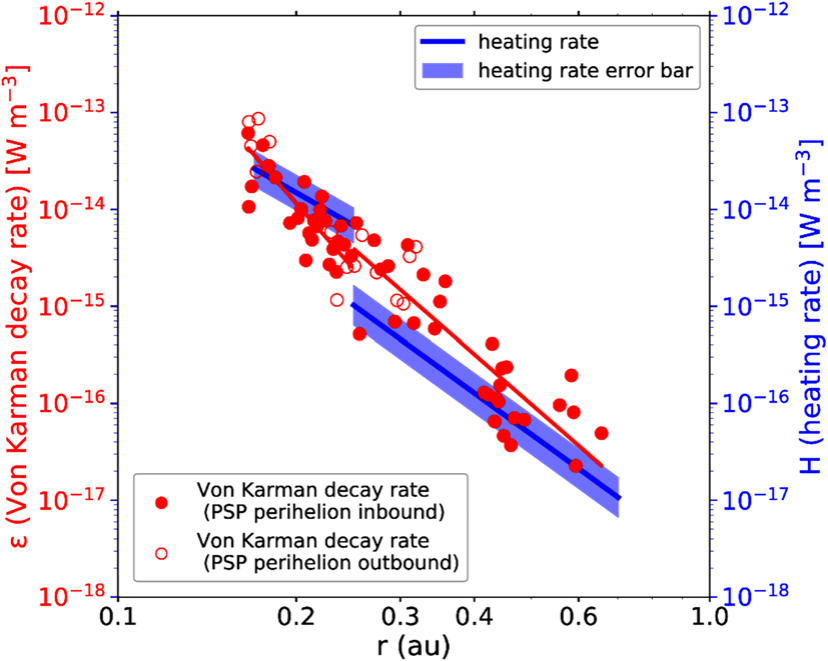
von Kármán decay rates compared to solar wind heating rates estimated from first three encounters of PSP. The red symbols represent decay rates and the lines and shaded region represent proton heating rates. (Reproduced with permission from Wu et al. (2022))

As seen in simulations (Wu et al. 2013; Parashar et al. 2015; Matthaeus et al. 2016; Shay et al. 2018) and in previous observations (Vasquez et al. 2007; Stawarz et al. 2009; Coburn et al. 2015), the von Kármán decay rate determines the rate of dissipation even in kinetic plasmas. PSP has already been used to test this balance in the inner heliosphere. Figure 14 shows the von Kármán decay rates and proton heating rates estimated using first three encounters worth of data (Wu et al. 2022). The red dots represent the von Kármán decay rate and the lines+shaded regions represent estimates of solar wind heating rates of protons (Tu and Marsch 1995; Wu et al. 2020; Martinović et al. 2020). The von Kármán decay rate is not only consistent with the energy supply rate estimated from the evolution of the large scale break frequency (see Fig. 3 of Wu et al. (2022)) but is also consistent with proton heating rate. This balance between the energy input rate and dissipation implies that the energy cascades in the inertial range down to kinetic scales in a conservative fashion.

### Energy in the inertial range

**Fig. 15:**
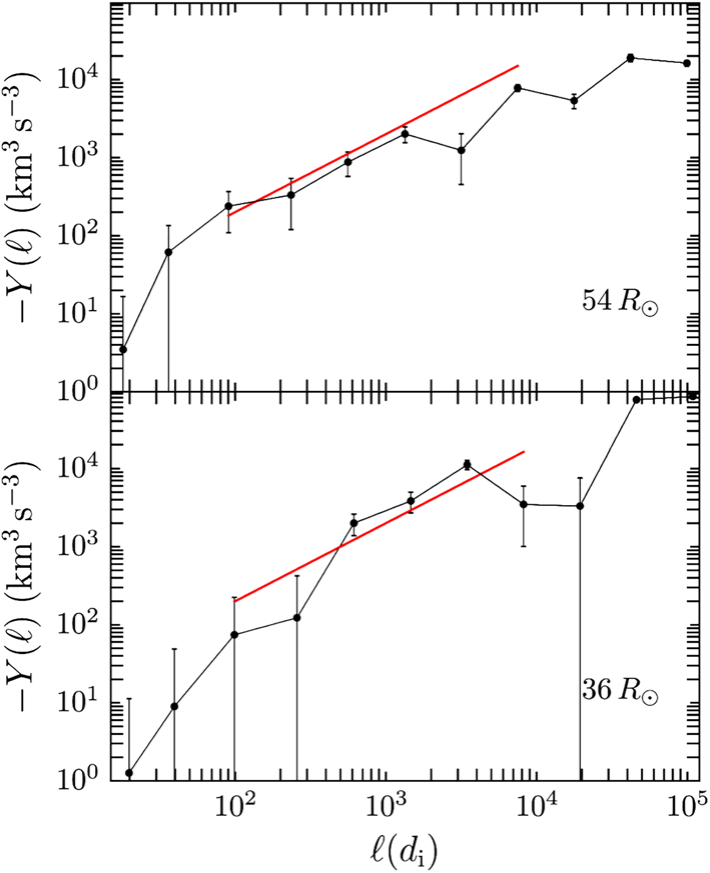
Incompressible Yaglom fluxes computed using PSP data from the first encounter Bandyopadhyay et al. (2020). The red lines represent fits used to obtain the cascade rate. (Reproduced with permission from Bandyopadhyay et al. (2020))

The energy input at the large scales cascades down to kinetic scales through the inertial range. This cascade rate can be quantified using the Politano Pouquet MHD generalization of Yaglom’s 3rd order law (see e.g. Eq. 2). Figure  15 shows two examples of the third order fluxes computed at two different radial distances using the incompressible version of the PP law. The red lines show linear scaling laws. These linear scalings, when identified, can be used to estimate the cascade rate in the inertial range (Bandyopadhyay et al. 2020). The cascade rates identified this way are compared with the von Kármán decay rates in Fig.  13 as squares with an orange line. The third order law decay rates are comparable to the von Kármán decay rates and are also comparable to simulation findings. A recent study compared the cascade rates in sub-Alfvénic wind with super-Alfvénic wind (Zhao et al. 2022). The cascade rate was computed to be higher in the sub-Alfvénic interval compared to the super-Alfvénic interval although longer sub-Alfvénic intervals would be needed to get statistically significant results. The cascade rates were also dominated by compressive terms compared to incompressive terms.

It is possible to estimate the decay rates from the isotropic and anisotropic versions of the PP law (see e.g. Podesta 2008 and other references in Sect. 2). Both isotropic and anisotropic cascade rates increase with decreasing heliocentric distance. This increase correlates well with the well established increase in fluctuation amplitude, Alfvénicity, and temperature with decreasing heliocentric distance (Andrés et al. 2022; Brodiano et al. 2022). The compressible cascade rates computed from PSP’s first encounter have been compared to compressible cascade rates at 1 AU (THEMIS data) and 1.6 AU (near Mars using MAVEN data). The compressible cascade rates show a drop by five orders of magnitude (Andrés et al. 2021) between 0.2 AU (PSP) and 1.6 AU (MAVEN). The density fluctuations are larger in the inner heliosphere. As the wind expands, it approaches a nearly incompressible state (Matthaeus et al. 1990; Adhikari et al. 2017). This change in the nature of solar wind turbulence could be related to the decrease in compressible decay rates.

**Fig. 16:**
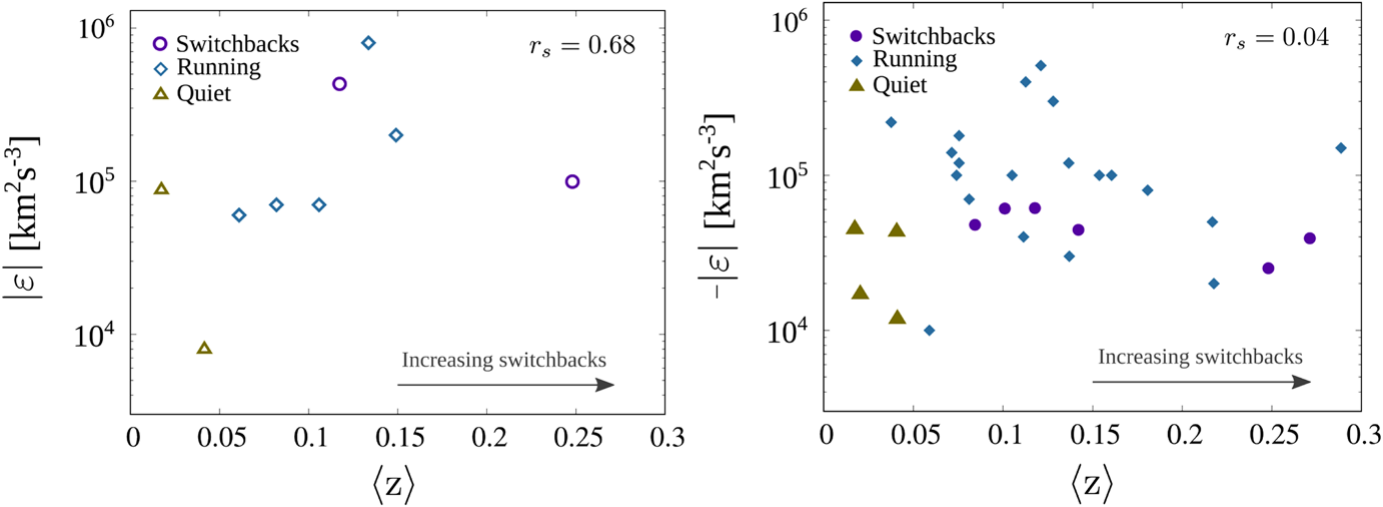
Cascade rates computed using Yaglom law inside and outside switchbacks Hernández et al. (2021). (Reproduced with permission from Hernández et al. (2021))

The presence of switchbacks can affect the cascade rates as well. Figure 16 shows the cascade rates computed in switchbacks identified during the first encounter of PSP (Hernández et al. 2021). Some switchback intervals show positive decay rate and some show negative decay rates. Although the interpretation of a negative cascade rate is unclear, it could imply a local transfer of energy to larger scales. The intervals that show a net positive cascade rate in the inertial rage show a visibly identifiable correlation with the switchback parameter *Z* (Hernández et al. 2021) (see, de Wit et al. 2020 for the definition of *Z* and detailed analysis of switchback properties). No such correlation is observed in cases when a negative cascade rate is identified in an interval (Hernández et al. 2021). The finding that the cascade rate is enhanced by the presence of switchbacks is consistent with the notion of more evolved turbulence in switchbacks (see e.g. Fig. 10). A local weak formulation of PP98 law gives a local in space and time dissipation function ${\mathcal {D}}$ (David et al. 2022). This local dissipation function also shows different behavior in and outside the switchbacks. It is predicted to follow $\sigma ^0$ scaling in the inertial range, $\sigma ^2$ in the viscous/dissipative range, and $\sigma ^{-1}$ at the discontinuities where $\sigma$ is the scale at which the local dissipation measure is computed. The dissipation measure ${\mathcal {D}}$, when averaged over the interval, shows behaviour similar to the third order law but locally it shows an unexplained scaling of $\sigma ^{-3/4}$ at the locations of switchbacks.

### Energy at kinetic scales

The cascaded energy is partially dissipated at the proton kinetic scales and part of it is cascaded down to electron scales. Various dissipative mechanisms and pathways have been proposed to explain dissipation in kinetic plasmas. These include wave-particle interactions such as cyclotron resonance (Hollweg and Isenberg 2002) and Landau damping (TenBarge et al. 2013), stochastic heating (Chandran et al. 2010; Martinović et al. 2021), and heating at intermittent locations (Parashar et al. 2011; TenBarge et al. 2013; Wan et al. 2016). Heating at intermittent locations can happen because of processes such as reconnection (Shay et al. 2018), and possibly because of Landau damping as well (TenBarge et al. 2013). A mechanism-agnostic approach focuses on the action of pressure strain interaction term, which also happens intermittently (Yang et al. 2017) (see Sect. 2 for a detailed discussion).

PSP’s state-of-the-art very high time cadence measurements in the inner heliosphere have enabled identification of various wave modes including circularly polarized waves, kinetic Alfvén waves, and electrostatic waves (Bowen et al. 2020b, 2020c; Verniero et al. 2020; Malaspina et al. 2020; Vech et al. 2021; Zhao et al. 2021; Cattell et al. 2021; Malaspina et al. 2022). The waves are found to last anywhere from a few seconds Bowen et al. (2020b) to “wave storms” a few hours long Verniero et al. (2022). Coincident with the ion-scale waves is a broadening of the beam of proton velocity distribution function (VDF) that is being termed as “hammerhead” of the proton VDF. It was also shown by Verniero et al. (2022) that these features are consistent with the expectations of quasilinear diffusion in velocity space in the presence of waves. A recent paper studies proton VDFs in the presence of cyclotron waves (Bowen et al. 2021). The VDFs show signatures consistent with quasilinear diffusion in the velocity space, indicating a possibility of cyclotron heating. This quasilinear diffusion is accompanied by steepening of magnetic power spectra to $\sim f^{-4}$ near proton scales. A potential explanation for such a combination of observations was recently proposed (Squire et al. 2022). It is suggested that a helicity barrier inhibits a cascade of energy to smaller scales (Meyrand et al. 2021). This results in a proton scale build up of energy and a steep transition range just below proton scales. This proton scale build up of energy in-turn generates ion-cyclotron waves, which can participate in preferential resonant heating of ions (Squire et al. 2022). This steepening of magnetic spectra at proton scales has also been used to estimate a dissipative removal of energy at proton scales. It was estimated that the power removed from the magnetic fluctuations at proton scales is sufficient to heat the solar wind (Bowen et al. 2020).

**Fig. 17:**
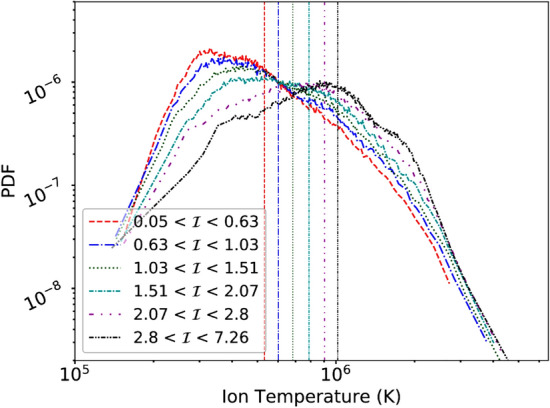
PDFs of temperature conditioned on PVI value. Vertical lines show the median temperature for each subset of the data. A clear rise of the median temperature with PVI is seen. (Reproduced with permission from Qudsi et al. (2020))

Another pathway to dissipation is stochastic heating (Chandran et al. 2010; Cerri et al. 2021) where particles experience stochastic kicks by the changing electric potential during their orbit (see section 2 for a discussion). The stochastic heating rate increases with decreasing heliocentric distance as $Q_\perp \propto r^{-2.5}$ (Martinović et al. 2021). It is larger in the fast wind compared to slow wind. Moreover, stochastic heating is enhanced inside the switchback regions. As mentioned before, stochastic heating enhances in the presence of intermittent structures. SBs show enhanced probability of finding large PVI values, and hence the turbulence in SBs is likely more intermittent. This could potentially enhance the stochastic heating rate. There is ample evidence for intermittent heating at 1 AU as well as in the inner heliosphere.

At 1 AU, the protons are observed to be hotter near strong PVI sites (Osman et al. 2011). Similar analysis in the inner heliosphere using PSP yields no new surprises. Figure 17 shows the probability distribution functions of temperature conditioned on PVI values. The probability of finding a higher temperature, say greater than $10^6$K, is higher for data conditioned on higher PVI values and the opposite is true for lower temperatures, say a few $10^5$K. This is also quantified by the increasing median value of temperature with increasing PVI value. Vertical lines in Fig.  17 show this systematic trend in the median temperature rise, indicating hotter populations for larger PVI values.

**Fig. 18:**
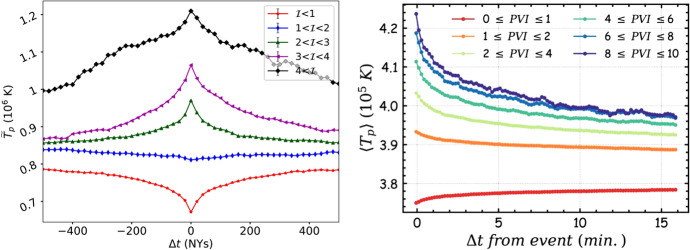
Conditioned temperature of protons near PVI structures (left: from second half of PSP’s encounter 1, right: from first six encounters’ worth of data). See text for details. Mean temperatures are higher near higher PVI values. (Reproduced with permission from Qudsi et al. (2020); Sioulas et al. (2022))

The proximity of dissipation and intermittent structures can also be quantified by computing the average temperature in the vicinity of PVI structures. This technique has been used to not only identify heating at intermittent structures (Osman et al. 2012), but also to show enhanced energetic particle fluxes near such intermittent structures (Tessein et al. 2013). The average is computed as $\tilde{T}_p(\Delta t, \theta _1, \theta _2) = \langle T_p(t_{\mathcal {I}}+\Delta t) |\theta _1< {\mathcal {I}} < \theta _2 \rangle$ where $\tilde{T}_p$ is the conditional average temperature for all events, $\Delta t$ is the lag relative to the PVI position, $t_{\mathcal {I}}$ is the location of PVI event, and $\theta _1$ and $\theta _2$ are the thresholds for selecting particular PVI values. Figure 18 shows conditional average temperatures for various PVI thresholds from the second half of PSP’s encounter 1 in the left panel and from the first six encounters in the right panel. Evidently, the temperature in the vicinity of larger PVI values is larger, even out to about a correlation length away, when compared to smaller PVI values. This analysis has also been used to show that the electrons show a similar behaviour (Phillips et al. 2022) and that the protons heat more, compared to electrons, in the vicinity of PVI structures (Sioulas et al. 2022). The fact that PVI values appear to be clustered in the inner heliosphere (Chhiber et al. 2020) could be one of the potential reasons behind consistently higher temperatures this far away from the intermittent PVI events.

Another possibility to study intermittent dissipation is to use LET, the local kernel of the third order law (Eqn. 2). Although LET varies significantly from one point to the other, its average at a given scale quantifies the net flow of energy into/out of that scale (Sorriso-Valvo et al. 2018). In a similar analysis to Fig. 18 using Helios data, the LET was shown to correlate better than PVI with the mean temperature even though there was shown to be a strong correlation between LET and PVI (Sorriso-Valvo et al. 2018). A potential reason behind the apparent lack of correlation between PVI and temperature in Helios data could be an artefact of the coarse resolution of Helios data which would not allow resolution of large small scale increments and hence the most intense PVI structures. PSP, with its fast measurements, allows resolution of PVIs at much finer scales and recovers the behaviour shown in Fig. 18.

**Fig. 19:**
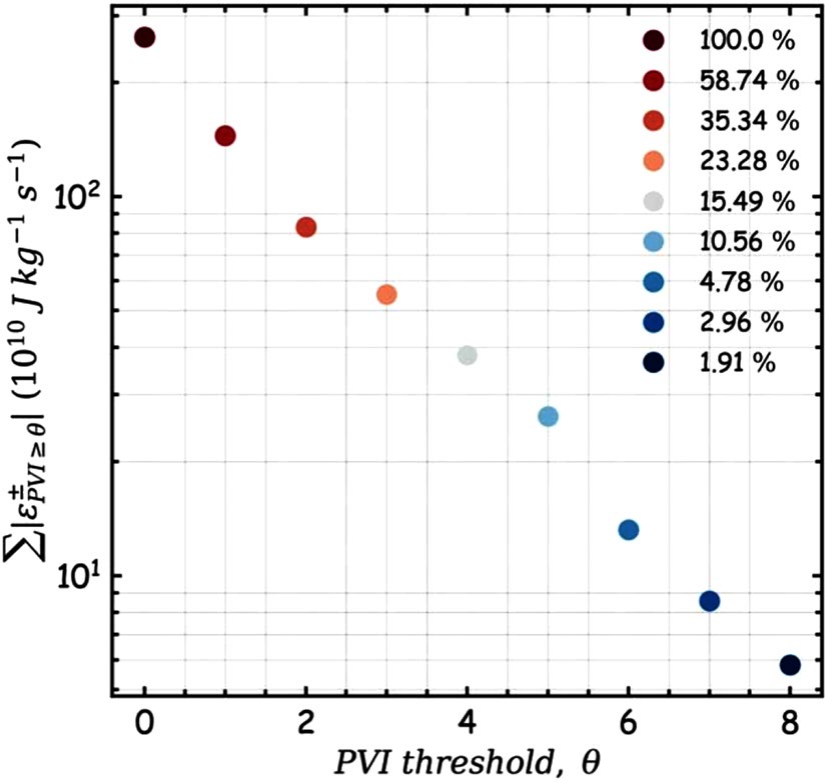
Dissipation measure contributions from PVI structures. The sum of the absolute value of the LET measure, conditioned over PVI decreases with increasing PVI threshold. PVI $>5$ contribute $\sim 11\%$ to the net dissipation Osman et al. (2012); Sioulas et al. (2022) (Reproduced with permission from Sioulas et al. (2022))

Larger PVI events occupy smaller fraction of the total volume but contribute significantly more to the budget of internal energy $U = C n k_B T$, where *C* is the specific heat capacity, $k_B$ is the Boltzmann constant, *n* the density, and *T* the temperature. At 1 AU, using ACE data, it has been shown that PVI $> 2.4$ occupy only 19% of the volume but contribute 50% to the internal energy budget, while PVI $> 5$ occupy only 2% of the volume but contribute $\sim 11\%$ to the internal energy budget Osman et al. (2012). Figure  19 shows the total absolute value of LET conditioned on PVI threshold from first six encounters of PSP (Sioulas et al. 2022). There are some similarities and differences in these findings when compared to Osman et al. (2012). Here PVI $>2$ contribute $\sim 35\%$ to the absolute LET measure, significantly less than what was estimated by Osman et al. (2012). On the other hand, PVI $>5$ contribute $\sim 11\%$ which is consistent with the findings of Osman et al. (2012). Evidently LET is a very different measure of dissipation compared to internal energy. The differences in the inferences of Sioulas et al. (2022) and Osman et al. (2012) could potentially stem from the different nature of these measures, or from other considerations such as enhanced possibility of wave damping (Bowen et al. 2021; Squire et al. 2022), or from yet largely unexplored clustering properties of the PVI events (Chhiber et al. 2020) or the transition of turbulence to monofractal behaviour at sub-proton scales potentially owing to scale invariance of current sheets in the kinetic range (Chhiber et al. 2021). Moreover, LET and PVI are scale dependent quantities. Similar studies with varied LET computation scale are required to explore such connections further.

## Summary and conclusions

The origins and evolution of the solar wind have been a mystery ever since its prediction and discovery (Parker 1958; Neugebauer and Snyder 1966; Tu and Marsch 1995; Marsch 2006; Bruno and Carbone 2013; Verscharen et al. 2019). Many iconic missions have enhanced our understanding of the processes that contribute to origins and evolution of the solar wind. However, the solar coronal dynamics were largely studied using remote observations until very recently. Parker Solar Probe is allowing the exploration of hitherto unexplored regions approaching and inside the Alfvén critical surface. Recently PSP entered the magnetically dominated solar corona with the perihelion at $15.9 R_\odot$ (Kasper et al. 2021). As PSP approaches closer to the sun, eventually reaching 9.8 $R_\odot$, it will allow exploration of various processes active in the solar corona, including the turbulence that potentially plays an important role in the heating of the Solar Corona.

In this paper we have focused primarily on the transfer of energy across scales in the inner heliosphere as observed by the Parker Solar Probe in the last four years. Rather than adopting a more detailed and mathematical *scale to scale* transfer approach (Verma 2019) that may not be amenable to spacecraft analysis, we adopt a more empirical approach based on the taxonomy of energy-containing scales, inertial range scales and kinetic scales. Based on such an approach, a significant amount of work has been done in the last four years on various aspects of solar wind properties and the turbulence. We refer the reader to [190] for a detailed discussion of many other aspects that were not covered here.

As the solar wind expands from inner heliosphere to the outer heliosphere, its turbulent nature changes dramatically (DeForest et al. 2016; Chen et al. 2020). Expanding from the solar corona, the wind becomes supersonic in the Alfvén critical region. Beyond the Alfvén critical region, the magnetic field loses control and velocity shears at flux tube boundaries can create flocculation, isotropizing the wind at the largest scales, and advancing the wind’s turbulent evolution (DeForest et al. 2016; Chhiber et al. 2018; Ruffolo et al. 2020).

The amplitudes of turbulent fluctuations decrease radially outwards while the outer scale gets larger. The outer scales increases with increasing heliocentric distance as a power law. The power-laws exponents found for outer scale vary between 0.5 and 1.5 whereas theoretical models predict the exponent to be generally less than 1 (Klein et al. 1992; Horbury et al. 1996; Ruiz et al. 2014; Bruno and Trenchi 2014; Chen et al. 2020; Wu et al. 2020). Intermittency increases from inner heliosphere up to 1 AU. A clear transition is seen in various properties at roughly 0.4 AU (Alberti et al. 2020; Cuesta et al. 2022; Alberti et al. 2022). Although it might appear that the bandwidth available for the turbulent cascade might keep increasing with increasing outer scale even beyond 1 AU, the inner scale (for example $d_i$) expands faster than the outer scale. This results in a reduced Reynolds number with increasing heliocentric distance. The reduction in the Reynolds number in-turn implies reduced intermittency, as quantified by radially decreasing small scale kurtosis and PVI values (Parashar et al. 2019; Cuesta et al. 2022).

The fluctuations are highly Alfvénic and imbalanced in the inner heliosphere with outward propagating fluctuations dominating the energy budget (Bavassano et al. 2000). The Alfvénicity drops as the wind expands with the dominant species ($\mathbf {z}^+$) decaying significantly faster than the minor species ($\mathbf {z}^-$) (Goldstein et al. 1995; Breech et al. 2008; Roberts et al. 1987; Matthaeus et al. 2004; Stribling and Matthaeus 1991). This is reflected in the decreasing cross helicity with increasing heliocentric distance as well.

Going sunwards, the enhanced fluctuation levels of velocity, magnetic field, as well as density imply that the energy input rate at the largest scales increases (Wu et al. 2022). The von Kármán Howarth decay rate increases by two orders of magnitude going from 1 AU to $\sim$0.2 AU (Bandyopadhyay et al. 2020). This enhanced decay rate balances estimated heating rates for the solar wind. In the inertial range, the incompressive decay rates compare favourably with the von Kármán Howarth decay rate and increase by a couple of orders of magnitude approaching the sun. The compressive component of the cascade rate could be significant in the inner heliosphere owing to the increased density fluctuations as well. It can decrease by as much as five orders of magnitude from 0.2 AU to 2 AU (Andrés et al. 2021), likely a consequence of the solar wind’s evolution towards a nearly incompressible state.

At the kinetic scale, the cascade is modified by kinetic physics as well as dissipation (Bowen et al. 2021). The spectral slopes in the imbalanced inner heliospheric turbulence routinely show a steep transition range with power law $\sim f^{-4}$ just below proton kinetic scales (Denskat et al. 1984; Leamon et al. 1999; Huang et al. 2021). This potentially happens because of enhanced heating of protons and an inhibition of the cascade to smaller scales (Meyrand et al. 2021; Squire et al. 2022).

The transfer of energy into internal degrees of freedom has been shown to happen via wave-particle interactions, stochastic heating as well as in intermittent locations (Vech et al. 2021; Martinović et al. 2019, 2020; Qudsi et al. 2020). Distribution functions in the presence of waves show signatures expected of quasilinear diffusion in the velocity space (Bowen et al. 2021; Verniero et al. 2022). Larger PVI values have been shown to associate favourably with hotter plasma, consistent with simulations as well as 1 AU observations (Qudsi et al. 2020). Local measures of dissipation have also been shown to relate with enhanced intermittent heating (David et al. 2022; Sioulas et al. 2022).

Many of the properties discussed above modify significantly in the presence of switchbacks. The turbulence appears to be more evolved inside switchbacks. The power spectra become Kolmogorov like inside SBs even when nearby NSB intervals show IK-like behaviour (Bourouaine et al. 2020). The stochastic heating rate, the incompressive cascade rate, and PVI values etc. are enhanced in the presence of SBs (Martinović et al. 2020).

Although PSP is already enabling a significant progress in studying turbulent transfer of energy across scales in the heliosphere, many open questions remain.

- How do we get true estimates of heating rates from single spacecraft measurements?
- Do such estimates modify the balance between the von Kármán Howarth decay rate and true heating rates?
- How do local dissipation measures (e.g. LET (Sioulas et al. 2022), ${\mathcal {D}}$ (David et al. 2022), field particle correlation (Verniero et al. 2021)) match with the large scale decay rates?
- What are the relative contributions of various cascade terms (incompressible, compressible, hall, etc.) to the inertial range estimates of decay rates?,
- How does this cascaded energy partition between ions and electrons? How much energy is taken away from the cascade by ion heating and how much is left to cascade down to electron scales?
- How does the cascade proceed closer to the electron scales?,
- Is there an identifiable relationship between intermittent dissipation and wave-particle interactions?
- How do the conclusions to above mentioned questions vary with ambient conditions?

Building upon the approximately six decades of research, PSP is allowing an in-depth exploration of turbulent regimes that were not accessible up to now, both in time/length scales and in proximity to the sun. With the abundance of switchbacks, identification of waves in turbulence, new proton VDF features, modified turbulence properties, and observations of turbulence in the sub-Alfvénic solar corona, PSP is only getting started. Over the next few decades, PSP will allow greater discoveries related to the origins of the solar wind, its evolution and the consequent turbulent properties.

## Footnotes

1 We use the terms ‘*dissipation*’ and ‘*heating*’ in a relaxed way to mean a transfer of turbulent fluctuation energy into the internal degrees of freedom of the plasma regardless of the (ir)reversible nature of the processes involved.
